# CRISPR-Cas9 Gene Editing of the *Sal1* Gene Family in Wheat

**DOI:** 10.3390/plants11172259

**Published:** 2022-08-30

**Authors:** Toni Mohr, James Horstman, Yong Q. Gu, Nagwa I. Elarabi, Naglaa A. Abdallah, Roger Thilmony

**Affiliations:** 1 USDA-ARS, Crop Improvement and Genetics Unit, Albany, CA 94710, USA; 2 Department of Genetics, Faculty of Agriculture, Cairo University, Giza 12613, Egypt

**Keywords:** CRISPR, wheat, *Sal1*, drought tolerance, *Triticum aestivum*, gene editing

## Abstract

The highly conserved *Sal1* encodes a bifunctional enzyme with inositol polyphosphate-1-phosphatase and 3′ (2′), 5′-bisphosphate nucleotidase activity and has been shown to alter abiotic stress tolerance in plants when disrupted. Precise gene editing techniques were used to generate *Sal1* mutants in hexaploid bread wheat. The CRISPR (Clustered Regulatory Interspaced Short Palindromic Repeats) Cas9 system with three guide RNAs (gRNAs) was used to inactivate six *Sal1* homologous genes within the Bobwhite wheat genome. The resulting mutant wheat plants with all their *Sal1* genes disabled had slimmer stems, had a modest reduction in biomass and senesced more slowly in water limiting conditions, but did not exhibit improved yield under drought conditions. Our results show that multiplexed gRNAs enabled effective targeted gene editing of the *Sal1* gene family in hexaploid wheat. These *Sal1* mutant wheat plants will be a resource for further research studying the function of this gene family in wheat.

## 1. Introduction

Wheat is a crucially important food source and one of the most widely grown crops in the world comprising approximately 17% of the world’s arable land and a global production of ~700 million tons (https://www.fao.org/3/y4011e/y4011e04.htm; accessed 30 May 2022). It provides on average 19% of the total calories in our diet and is one of the most important sources of plant protein. Bread wheat is unique among cereal crops in that it contains gluten which enables the production of leavened bread and is a major ingredient in numerous other foods [1].

The gap between supply and demand in wheat, being a strategic commodity, makes it imperative to increase production overall which includes areas where suboptimal conditions prevail, i.e., water deficit, salinity and high temperature. As irrigation water sources have become scarcer, the development of crop cultivars with improved adaptation to drought is a major goal in wheat improvement programs. Global climate change has resulted in a general warming and the occurrence of more frequent instances of drought and other abiotic stresses worldwide becoming a major threat to international food security, as it reduces agricultural yields by up to 50% [2,3]. The development of drought tolerant wheat cultivars is an important challenge and necessary to ensure future food security [3,4,5,6]. Drought is a complex trait, which has made the development of drought tolerant wheat cultivars challenging, but new technologies are providing novel opportunities for the genetic improvement of wheat [4,5].

An efficient genome editing system called Clustered Regulatory Interspaced Short Palindromic Repeats, with the associated Cas9 nuclease (CRISPR/Cas), has emerged as a powerful tool for the precise manipulation of eukaryotic genomes including those of important crop plants [7,8,9,10,11,12,13,14]. The CRISPR/Cas system is an RNA directed tool for making precise breaks in target DNA sequences. These breaks are repaired by non-homologous end joining, which can introduce errors of small insertions or deletions within the target sequence. Thus, the CRISPR/Cas system is an efficient way of generating targeted gene knock-outs [7,9,14]. The sequence specific targeting of the Cas9 nuclease is provided by the binding of a small synthetic RNA referred to as a single guide RNA (gRNA). Since its discovery, the CRISPR/Cas system has been shown to efficiently mediate targeted double stranded breaks in a wide array of species including plants [7,8,11,12,13,14].

The CRISPR/Cas system was initially shown to efficiently mediate targeted double stranded breaks in model plants such as *Arabidopsis* and tobacco [15,16], but its utility in important crop species like rice and wheat soon followed [12,13,14,17,18,19,20,21,22,23]. The ability of this system to efficiently generate targeted mutations is particularly useful in crops like wheat which contain very large and complex genomes. The use of this system in discovering gene functions and potentially engineering abiotic/drought stress tolerance or signaling in wheat will likely prove useful in creating novel genotypes with desirable traits in the future [12,13,14,24].

The *Sal1* gene encodes a bifunctional enzyme with inositol polyphosphate-1-phosphatase and 3′ (2′), 5′-bisphosphate nucleotidase enzyme activity that is hypothesized to be a plastid-localized oxidative stress sensor [25,26,27,28,29]. Research performed in *Arabidopsis* has shown that mutant plants lacking a functional *Sal1* (also known as *FRY1*, *HOS2*, *ALX8*, *OLD101*), exhibit drought stress tolerance [25,26,27,28,29,30]. The *Sal1* plants exhibit a reprograming of their metabolism resulting in an increase in osmoprotectant compounds and the stress hormone abscisic acid, which manifests itself as abiotic stress tolerance [27,28,29]. The *Sal1* gene also has been implicated in controlling reactive oxygen species levels and leaf senescence in *Arabidopsis* [30]. The transient silencing of *Sal1* expression in wheat using a viral-based expression system has suggested that wheat lacking or with reduced Sal1 activity also exhibits drought tolerance under short-term water stress [31].

To investigate the role of *Sal1* in hexaploid bread wheat, CRISPR gene editing was used to introduce mutations within the gene family. The generation of transgenic Bobwhite wheat plants, their molecular characterization including the identification of targeted mutations and the characterization of the growth, morphology and response to abiotic stress in the mutants is presented.

## 2. Results

### 2.1. Sal1 Gene Family in Wheat

*Sal1* is a highly conserved single copy gene in the genomes of the model plants *Arabidopsis thaliana* and *Brachypodium distachyon* [32]. Modern bread wheat, however, is a recent hexaploid undergoing rapid and complex genome evolution. We performed bioinformatics analyses using the publicly available genome sequences to investigate the number and functionality of *Sal1* genes in wheat. 

In Chinese Spring, preliminary analysis found seven different homeologs of *Sal1*: 5ABD, 7AD, and two genes on 4A (4A-1, 4A-2). The pair of genes on 4A are less than 50 kilobase pairs (kb) apart and are highly similar except for a single base pair deletion in the seventh exon that causes a frameshift and premature stop codon in the 4A-1 allele. Although this is near the end of the coding sequence, the high degree of evolutionary conservation in this region implies that 4A-1 may be a pseudogene. All other *Sal1* homeologs appear to encode a functional protein sequence. 

To investigate the evolution of this small gene family in other wheat varieties, a 203 base pair conserved section of the Chinese Spring *Sal1* 5D allele covering conserved amino acids in the fourth exon was BLAST searched against several other available wheat genomes (Table 1). The *Aegilops tauschii* D genome carries two *Sal1* alleles present on chromosomes 5 and 7. In tetraploid wheat (*Triticum turgidum,* AABB), the uncultivated subspecies *dicoccoides* (accession Zavitan) has three copies on chromosomes 7A, 5A, and only a single functional copy on 4A (4A-2), while the cultivated subspecies *durum* (cv. Svevo and Kronos) also have 5B and the 4A-1 genes. *Triticum spelta* (accession PI190962) and *Triticum aestivum* cv. Norin 61 have the same seven alleles as Chinese Spring, however, most other cultivars of *Triticum aestivum* have lost the 5A homeolog of *Sal1*. The recently bred Canadian cultivars CDC Landmark and CDC Stanley have five *Sal1* homeologs; they are missing 4A-2 and instead have a 4A-1 homeolog that does not have the single base pair deletion found in other wheat genomes.

An investigation of publicly available gene expression data in Genevestigator found that all of the wheat *Sal1* homeologs have expression data except 7D, which was not annotated in the available datasets. Moreover, 5D is the most highly expressed gene and 5A has the lowest expression, with others being intermediate (Figure S1). The low apparent levels of 5A expression in the datasets is likely due to the fact that the homeolog is missing in many wheat cultivars.

To characterize the gene family within the Bobwhite genome, the *Sal1* genes were PCR amplified using primers based on conserved sequences within the gene family present in Chinese Spring (Table S1). The amplified products were cloned and individual clones were sequenced (Figure S2). Although most of the homeologs were easily amplified, a 5A *Sal1* gene was not amplified from Bobwhite, suggesting that this cultivar also lacks this homeolog like several other wheat cultivars (Table 1). The sequence analysis also showed that the cloned Bobwhite *Sal1* sequences were highly similar to the Chinese Spring reference genome. Only a few nucleotide polymorphisms were observed in Bobwhite, resulting in four modest amino acid changes in the encoded *Sal1* proteins. An alignment of the Bobwhite *Sal1* protein partial sequences with the *A. thaliana* and *B. distachyon* sequences also shows a high level of conservation between Bobwhite and these model species (Figure S3).

### 2.2. Design and Transformation of the CRISPR-Cas9 Sal1 Knockout Construct

**Figure 1:**
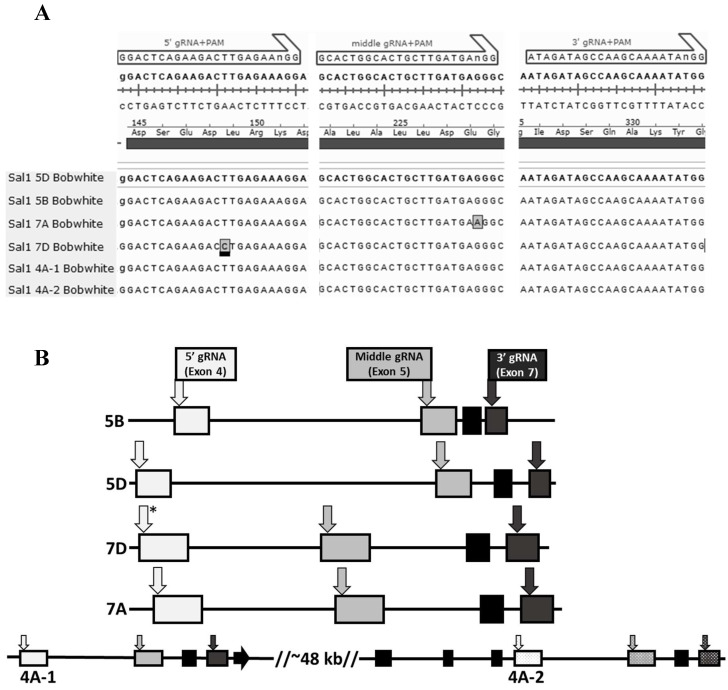
CRISPR targeted regions in the wheat *Sal1* gene family. (**A**) Conserved regions of wheat *Sal1* genes targeted by CRISPR editing. The underlined boxed residue indicates a 1 basepair mismatch in the *Sal1^7D^* 5′ target site sequence. (**B**) Diagram showing the location of the target sites in the six Bobwhite *Sal1* genes. Black lines indicate introns and boxes denote exons. The *Sal1^4A−1^* and *Sal1^4A−2^* genes are in tandem on chromosome 4A, separated by approximately 48 kilobases (kb). The asterisk indicates the mismatch to the *Sal1^7D^* 5′ target sequence.

To maximize the probability of creating complete *Sal1* knockout hexaploid wheat plants, three single guide RNAs (gRNAs) (Table S2) targeting conserved nucleotide sequences within the wheat *Sal1*-like genes were designed using the CRISPR MultiTargeter web tool [41]. These target sequences are almost completely conserved within the Bobwhite *Sal1* alleles (Figure 1A), with only a single functional mismatch seen in the 7D 5′ target. We hypothesized that since *Sal1* is highly conserved in plants [32], mutations introduced within the exon 4, 5, and 7 target sites (Figure 1B) will likely cause amino acid changes or frameshifts producing a nonfunctional protein.

In the *Sal1* targeting construct, the guide RNAs are flanked by transfer RNA (tRNA) spacers and are expressed using the switchgrass *Ubiquitin1* (*PviUbi1*) promoter with the nopaline synthase (*nos*) transcription terminator [42]. The *Cas9* gene is constitutively expressed under the control of the maize *Ubiquitin 1* promoter (*ZmUbi1*) and *nos* terminator (Figure S4). The *Sal1*-targeting CRISPR construct was combined with a second plasmid carrying the *bar* plant selection marker gene which confers resistance to the herbicide bialaphos and biolistically transformed into Bobwhite. A total of approximately 3000 immature embryos were bombarded in multiple independent experiments over a period of more than 6 months. Genomic DNA was extracted from the regenerated herbicide tolerant wheat plants and the presence of the *Cas9*, *bar* and *PviUbi1* promoter sequences were confirmed with PCR amplification in 32 independent transgenic events (screening primers are shown in Table S2).

### 2.3. Identification of Wheat Sal1 Mutant Lines

**Figure 2:**
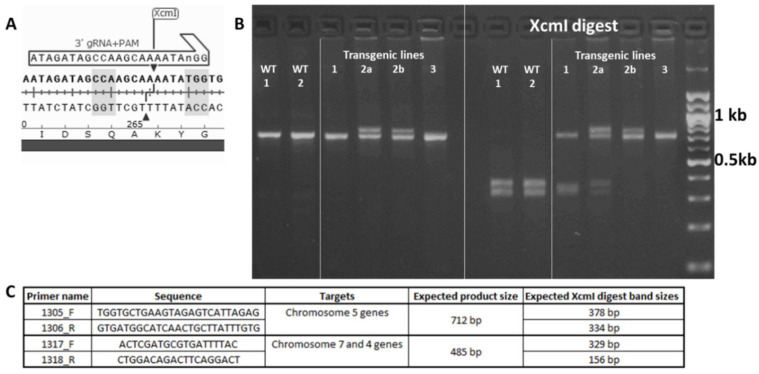
Screen to identify targeted *Sal1* mutations. (**A**) Location of the *Xcm*I cut site within the 3′ gDNA targeted region (the shaded CCA………TGG nucleotides denote the recognition site). (**B**) Agarose gel electrophoresis showing the PCR product of the chromosome 5 screening primers in wildtype Bobwhite (WT) and mutant transgenic lines before and after *XcmI* digest. Wildtype PCR product is completely cut by *XcmI,* whereas those with mutations in the 3′ site show larger and/or uncut bands. Transgenic lines 2a and 2b are sibling lines that inherited the same 56 bp DNA insertion into the 3′ site of 5D, which appears here as a larger band in the PCR assay. (**C**) Primers used in PCR amplification of the 3′ target regions screened in the assay.

The *Sal1* 3′ gRNA target site contains an endogenous *Xcm*I recognition site enabling the use of a Cleaved Amplified Polymorphic Sequences (CAPS) assay to identify introduced mutations (Figure 2A). The recognition site for *Xcm*I is four base pairs upstream and downstream of the likely Cas9 double-stranded break site, so most small insertions, deletions and some targeted point mutations will prevent *Xcm*I digestion (Figure 2B). Two pairs of primers were designed to PCR amplify the sequence spanning this region: one for chromosome 5 homeologs, and one for chromosome 7 and 4 homeologs (Figure 2C). The CAPS assay was used to screen for mutations within the 32 independent transgenic T_0_ events. The results identified seven events with mutations that blocked *Xcm*I digestion (15.6% of the independent transgenic events) and five of these events generated T_1_ progeny where the mutations were inherited. Three events carried mutations within both CAPS assays (suggesting mutation in multiple *Sal1* homeologs). These selected events were then propagated to the T_2_ and T_3_ generations for further characterization (Tables S4 and S5)**.**

One of the heritable events appeared to be a chimeric T_0_ plant, since the T_1_ progeny were a mix of wildtype and mutant plants based on CAPS screening. The T_2_ progeny from one of these wildtype T_1_ plants were confirmed as nontransgenic and nonmutant in the *Sal1* target sites and were subsequently used as a tissue culture/regeneration control (RC) in phenotyping experiments.

Using the CAPS screening assay, three independent events were identified as having complete or nearly complete mutations within the 3′ target sites. These events also appeared to have multiple transgenic loci, since no nontransgenic offspring were recovered in subsequent generations. Two individuals from independent events, hereafter referred to as 6KO1 (6 Knock-Out 1) and 6KO2, carried mutations in every sequenced *Sal1* target site, with the exception of the 7D site that contained a single mismatch with the 5′ gRNA. Several major rearrangements in these two events included the insertion of transgenic DNA (Figure S5). The incorporation of transformation construct fragments into *Cas9* target sites has been described before in wheat, potato, and biolistically transformed rice [43,44,45]. An unusual mutation found in two independent events was the chimeric fusion of the two 4A alleles, indicating a deletion of an approximately 48 kb region that was located between the 4A alleles (Figure S5A). These mutations likely occurred soon after bombardment (Milner et al. 2020), which is consistent with the observation that a portion of the transformation construct integrated within the target locus. Many of the mutations detected at the other target sites were small insertions/deletions (indels) or were unamplified with multiple different primer pairs, suggesting either a large deletion or rearrangement.

A third independently transformed event had several missing regions in the *Sal1* 5B and 5D alleles, but otherwise carried small indels in most target sites. The T_0_ plant was notably smaller and less fertile than other transformants, possibly as the result of somaclonal variation or the presence of unique mutations. Unlike 6KO1 and 6KO2, not all *Sal1* target sites were mutated in this line; however, all sequenced genes appear to produce a nonfunctional protein. The only mutation in this line that did not result in a frameshift occurred within 4A-1 (which may be a pseudogene) and resulted in the loss of a single highly conserved amino acid that may also compromise function. This mutant line was called MM (for multiple mutant).

The two other independent transgenic events appeared to have a single transgenic locus, as they had an approximate 3:1 segregation of transgenic offspring. Several of these offspring inherited several *Sal1* mutations (based on the CAPS assay screening), but were herbicide sensitive and lacked detectable transgenes. As *Cas9* was no longer present to create further mutations, we used these lines to investigate the effect of specific mutations within this portion of the *Sal1* gene family. These nontransgenic *Sal1* mutant lines were named PM1 (Partial Mutant 1) and PM2. PM1 has two different mutations in the 7A alleles and the 4A-1 gene targets could not be amplified. The PM2 event was missing both the 4A-1 and 4A-2 target sites and the 7D gene had a 1 bp insertion within the 5D 3′ target site. Thus, the 7A and 5B genes were the only unmutated homeologs in PM2, while PM1 had intact 5B, 5D, 4A-2, and 7D *Sal1* genes (Table 2 and Table S4). 

To further characterize the mutant lines, we designed qPCR primers and analyzed the expression of *Sal1*. Due to the high degree of sequence conservation in this gene family, we were unable to design primers that were homeolog-specific; however, a primer pair that was specific for chromosome 5 genes and a primer pair specific for the chromosome 7 and 4 genes were developed. The results from this analysis show that negligible levels of *Sal1* transcripts were detected in our 6KO mutant lines compared to the control lines (Figure S6). This result is consistent with the conclusion that the 6KO1 and 6KO2 mutants are full knock-out lines that are unable to express a functional *Sal1* gene.

### 2.4. Morphological Characterization of the Sal1 Mutant Plants

**Figure 3:**
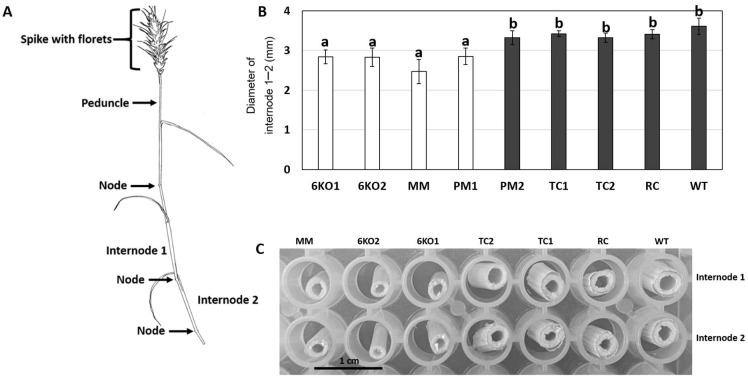
Stem phenotype in *Sal1* mutant Bobwhite plants. (**A**) Wheat stem diagram with labelled parts. (**B**) Width (mm) of internodes 2 and 3 of the primary stem of well-watered plants (see Table 2 for descriptions of each line). Letters show statistically significant differences (*p* < 0.01; n = 5). (**C**) Image of stem cross-sections of internodes 1 and 2.

Wheat plants were grown in the greenhouse to the T_3_ generation for most lines (T_2_ for the PM2 line) and their growth was measured and monitored during development (Table 2). To ensure that our observations of the mutant lines were not biased by phenotypic changes induced by the transformation and/or tissue culture process, we included a variety of control lines including two transgenic control lines (TC1 and TC2) (which were transgenic but had no detected *Sal1* mutations,) and a regeneration control line (RC, a nontransgenic, nonmutant line derived from a chimeric T_0_ plant described in Table S4).The 6KO, MM and PM1 lines were observed to have stems that were smaller in diameter than the control lines (Figure 3). This was most dramatically and consistently observed in the internode region between the first and second nodes of the primary tiller. Interestingly, this phenotype was not present in any of the control lines (RC, TC1, TC2, WT) or PM2. It was also consistently observed in all of the T_3_ 6KO1, 6KO2, MM and PM1 plants, so the causative mutant gene(s) were not segregating in these plants. Given that PM1 only has target mutations in the 7A and 4A-1 genes, and the PM2 line lacks the phenotype (and has a fully functional 7A homeolog, but a mutant 4A-1 gene), this suggests that the *Sal1* 7A homeolog may have a role in stem growth and morphology in Bobwhite wheat. Although this developmental phenotype was consistently observed (Figure 3B,C), we did not observe a significant difference in the plant height, leaf number, tiller number, tiller height, or reproductive tiller number between the *Sal1* mutants and the control lines.

### 2.5. Characterization of the Stress Tolerance of the Sal1 Mutant Plants

**Figure 4:**
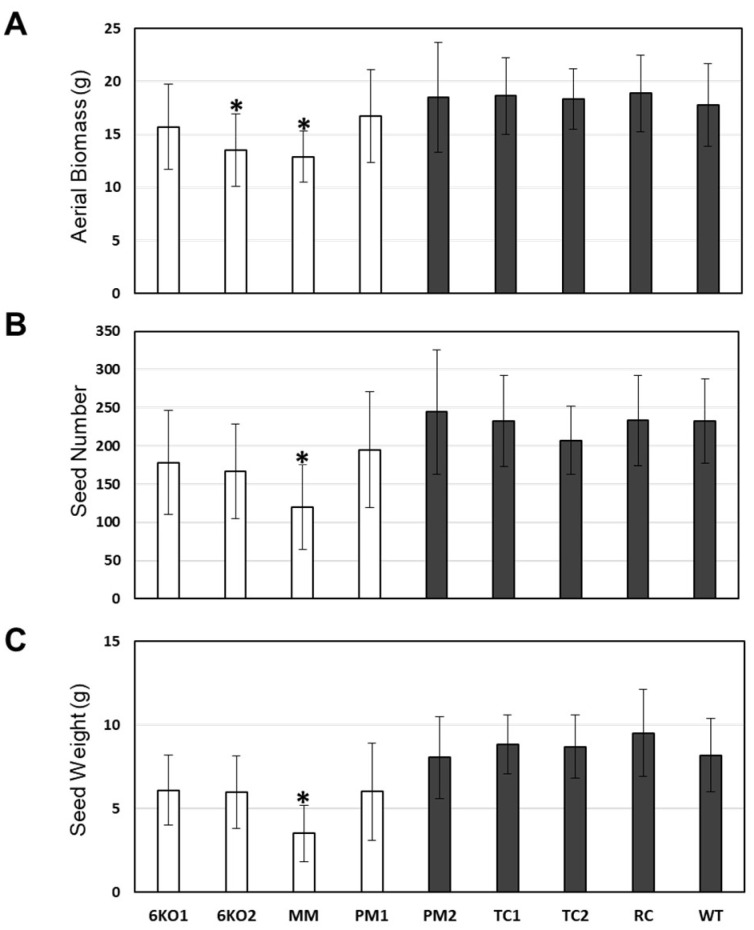
Growth of well-watered wheat lines. (**A**) Average weight of dry aboveground biomass (g), (**B**) seed number and (**C**) total seed weight (g) per plant. Results averaged from 3 independent experiments. * indicates statistically significant differences from controls (*p* < 0.05; n = 14). White bars indicate plants with mutant stem phenotype; dark grey bars indicate plants with wildtype stems.

A variety of experiments were also performed to investigate whether the *Sal1* mutants grew differently under various drought stress and well-watered conditions. This included mild drought stress or iron deprivation using germinated seedlings in “cigar rolls” of germination paper, mild to severe salt stress during germination using up to 200 mM NaCl in MS media, germination on up to 15% polyethylene glycol-infused MS media, and germination on media supplemented with the plant hormones abscisic acid and gibberellic acid. Significant differences were not observed between control seedlings and any of the *Sal1* mutant seedlings (data not shown). Both the mutant and control lines also grew similarly well under well-watered conditions (Figure S7) and were not significantly different for most of the measured traits (Figure 4). However, we did notice that the mutant lines with the stem phenotype (6KO1, 6KO2, MM, and PM1) appeared spindly compared to the control lines, and two lines (6KO2 and MM) produced slightly less aboveground biomass (Figure 4A). The control lines in these experiments all grew consistently well and were statistically indistinguishable. The MM line also had significantly lower seed number and overall seed weight under these well-watered conditions (Figure 4B,C).

**Figure 5:**
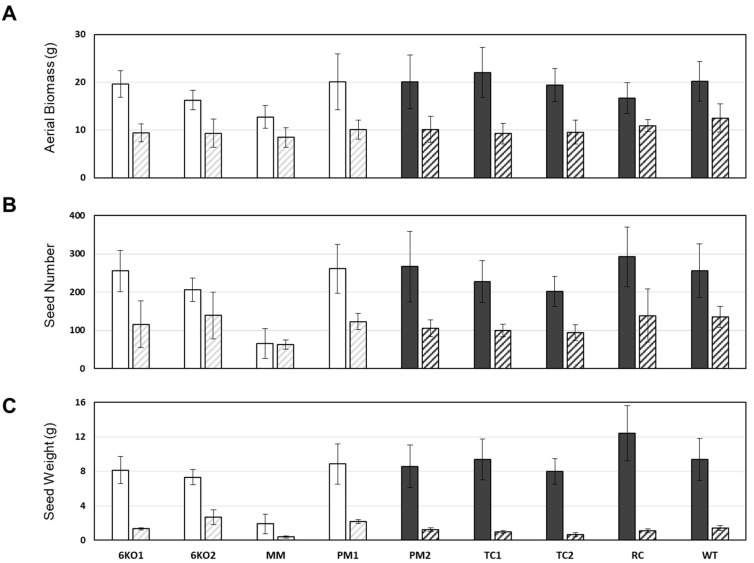
Comparison of growth in well-watered plants (solid) vs mild drought treatment (diagonal pattern) per plant: (**A**) aboveground dry biomass (g), (**B**) seed number, (**C**) total seed weight (g). White bars indicate plants with mutant stem phenotype; dark grey bars indicate plants with wildtype stems. Error bars indicate standard deviation (n = 4). Data is representative of 3 independent experiments.

Drought in the field most often occurs during wheat flowering and seed development, so we examined the performance of the *Sal1* mutants under terminal drought conditions by withholding additional water from the pots 5.5 weeks after planting. All experimental plants were hand-watered to full soil capacity at the start of this treatment to minimize differences in water content between pots. Under these conditions, the *Sal1* mutant lines (particularly 6KO2 and MM) stayed green longer than the control lines and the soil in their pots appeared to dry out more slowly (Figure S7D). All lines had reduced biomass, seed number, and seed weight under these conditions compared to well-watered plants, except MM had similarly low seed number in both conditions (Figure 5). These results suggest that the Bobwhite *Sal1* mutant plants do not exhibit improved yield compared to wildtype plants in these water limiting conditions. Porometry measurements were taken over the course of three weeks during this experiment, and although the drought-treated plants had an expected lower stomatal conductance than the well-watered plants, no significant differences were observed between the controls and various mutant lines (data not shown).

**Figure 6:**
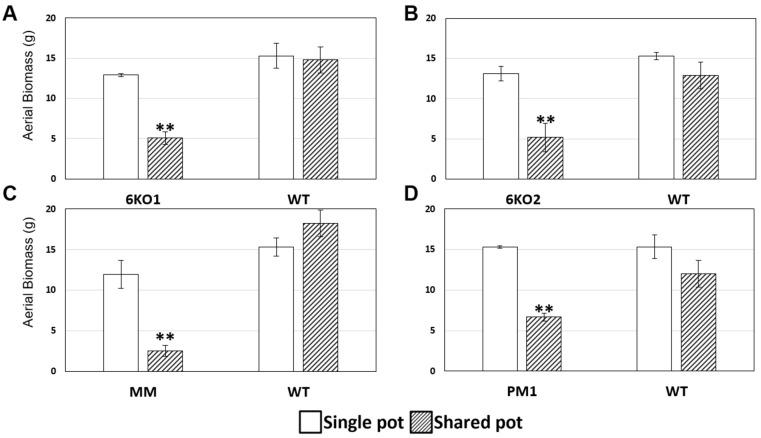
Growth of well-watered wildtype (WT) and mutant lines in single pots (white) and shared pots (diagonal pattern). The average dry aboveground biomass (g) for wildtype and (**A**). 6KO1 (**B**). 6KO2 (**C**) MM and (**D**). PM1 mutant plants. ** = *p* < 0.01; n ≥ 3.

To further interrogate the physiology of the *Sal1* mutant lines, their growth was examined when grown in the same pot as an equal number of wildtype Bobwhite plants, both under well-watered and drought conditions. Under these conditions the plants within the pot are competing for the same resources and should be subjected to the same degree of water stress. The 6K01, 6KO2, MM and PM1 lines co-grown with wildtype Bobwhite in a shared pot had significantly less aboveground biomass than when grown in their own pot, while Bobwhite’s biomass levels were unchanged in shared or single pots (Figure 6). This growth differential, not surprisingly, also extended to seed number and seed weight of well-watered plants (Figures S8 and S9) and aboveground biomass of drought-treated plants (Figure S10). The *Sal1* mutants also had less biomass in drought-treated shared pots than drought treated single-line pots (Figure S10).

## 3. Discussion

Creating gene knockouts is far more difficult in large, complex polyploid genomes such as bread wheat than in plant model systems. The multiple-targeting CRISPR-Cas9 gRNA strategy we used was successful in creating a variety of heritable *Sal1* mutations in several independent wheat lines. The complexity of mutations was potentially accentuated by the use of biolistics for transformation, resulting in more transgenic DNA fragments available for incorporation in genome breaks. However, this strategy also created mutations that allowed us to investigate several partial and complete mutants without having to extensively screen several generations of plants. It is also interesting to note that no mutations were identified in the one target site which contained a mismatch to the gRNA sequence used (the 5′ site of *Sal1* 7D), which is an encouraging sign that CRISPR-Cas9 sequence recognition in the research presented was still highly specific.

The narrow stem phenotype observed in several of the recovered mutants implies that *Sal1* 7A may have an important role in stem growth in Bobwhite. Interestingly, although *Sal1* 7A is not strongly expressed in shoot apical meristem compared to some of the other homeologs (Figure S1), and its core protein sequence is almost identical to the other homeologs in bread wheat (Figure S2), it appears to be responsible for the observed stem phenotypic differences. Further research would be required to definitely determine whether *Sal1* 7A has a unique role in stem growth, or if its loss simply alters the overall amount of *Sal1* activity in the developing tissues. Although we did not notice any root development differences in the *Sal1* mutant seedlings, the differences in aboveground biomass could also potentially extend to root biomass as well. However, the possibility of unique functional roles in such a highly conserved gene family offers potentially interesting insights into the function of *Sal1* homeologs in polyploid genomes.

Our *Sal1* mutant plants were slightly smaller and exhibited delayed senescence under drought conditions. This is consistent with an earlier study on drought stress resistance using virus-induced gene silencing in wheat, which described that plants with *Sal1* silenced were smaller than controls or lines silenced with other constructs [31]. However, smaller plants use less water during a drought experiment than larger plants, which can lead to a false impression of “drought stress resistance” [46].

For this reason, we conducted our drought studies using terminal conditions similar to those found in the field, where improved quantity and/or weight of seeds is the primary measure of successful drought stress resistance. Unfortunately, we did not see an improvement in the yield of mutant plants compared to that of control lines. The reduction in growth when plants were grown together in shared pots with wildtype Bobwhite indicates that the mutant plants were unable to compete for their share of resources, indicating that full *Sal1* knock-outs are deleterious in Bobwhite wheat. 

However, given the complexity of *Sal1* evolution in the wheat family, we cannot rule out that a specific gene deletion may lead to favorable abiotic stress tolerance phenotypes in different cultivars or genetic backgrounds. The variation of the number of *Sal1* alleles between wheat cultivars suggests that the role of any given family member must be experimentally determined.

## 4. Materials and Methods

### 4.1. Identification, Cloning, and Sequencing of Bobwhite Wheat Sal1

Bioinformatics analysis of the publicly available wheat genome sequence initially revealed seven *Sal1* homologs in the *T. aestivum* reference Chinese Spring genome by BLAST searching with the *Arabidopsis* protein sequence. Analysis of publicly available expression data (RNAseq data from Genevestigator) showed that at least six of the seven alleles were expressed; 7D was not annotated as a gene and there is no evidence of its transcription. The expression data (Figure S1) was accessed from Genevestigator (www.genevestigator.com) on 19 April 2022. A 216 base pair fragment of the *T. aestivum* Chinese Spring *Sal1* 5D allele covering Exon 4, including the 5′ gRNA target site, was used to interrogate the high-quality wheat genome sequences available at GrainGenes (https://wheat.pw.usda.gov/blast; accessed 11 April 2022). This region is highlighted in the Bobwhite *Sal1* 5D sequence shown in Figure S2. 

Genomic DNA was extracted from Bobwhite wheat leaves using a modified Puregene kit protocol (Qiagen, Redwood City, CA USA). Chinese Spring genomic sequences were used in Primer3 (https://primer3.ut.ee/; accessed 5 October 2021; Table S1) to design allele-specific primers for *Sal1* covering the three CRISPR/Cas9 target sites [47]. PCR products were amplified with the Q5 proofreading DNA polymerase (New England Biolabs, Ipswich, MA, USA) and amplicons of the expected sizes were gel-purified with the Zymoclean Gel DNA Recovery kit (Zymo Research Inc., Irvine, CA, USA), ligated overnight with T4 ligase into a StuI-digested pUC-Blunt plasmid vector (New England Biolabs, USA), transformed into chemically competent E. coli NEB10β cells (New England Biolabs, USA) and plated onto solid LB media with 100 mg/L carbenicillin. Plasmid DNA was isolated with the ZR Plasmid Miniprep kit (Zymo Research Inc., USA) and sequenced with the M13 forward and M13 reverse, and additional internal gene-specific primers when needed. One or more pairs of allele-specific primers were used to investigate mutant *Sal1* alleles and a potential Bobwhite 5A homeolog (which was not detected). Sanger sequence reads were aligned with the Chinese Spring sequence and annotated using Snapgene (www.snapgene.com; accessed 5 October 2022). 

For the *Sal1* protein sequence alignment (Figure S3), *T. aestivum* Bobwhite, *B. distachyon* (Bradi4g40860), and *A. thaliana* (AT5G63980) proteins were manually trimmed to remove the regions that were not sequenced in the Bobwhite *Sal1* homeologs. The protein alignment was performed using Clustal Omega (CLUSTAL O (1.2.4)) at https://www.ebi.ac.uk/Tools/msa/clustalo/ (accessed 11 April 2022) with the default parameters. Note that the *Sal1* 5B sequence had a gap starting at residue 60, since we were unable to amplify and sequence that portion of the Bobwhite 5B sequence, likely due to the presence of a large intron nearby that contained a sequence we could not amplify across.

### 4.2. CRISPR Sal1 Transformation Construct

The CRISPR *Sal1* transformation construct was constructed using standard restriction enzyme cloning techniques. The map and sequence of the plasmid is shown in Figure S4. The construct carries three *Sal1*-targeting guide RNAs (gRNA 5′, gRNA middle, and gRNA 3′) flanked by transfer RNA (tRNA) spacers, which are expressed using the switchgrass *Ubiquitin 1* promoter and the nopaline synthase transcription terminator. The *Cas9* gene is constitutively expressed in wheat using the maize *Ubiquitin 1* promoter and *nos* transcriptional terminator.

### 4.3. Bobwhite Wheat Biolistic Transformation

Transformation experiments were performed with a mixture of the CRISPR transformation plasmid construct and the pAHC20 plasmid which carries the maize *Ubiquitin 1* promoter and *nos* terminator controlling the expression of the *bar* selection gene which confers resistance to the bialaphos herbicide [48]. Bobwhite spring wheat transformation was performed using a standard biolistic transformation method as previously described [49,50,51]. In brief, 1µm gold particles were coated with a 1:1 mixture of the CRISPR and pAHC20 plasmids. The coated particles were bombarded into immature dissected wheat embryos using the PDS-1000 Helium Gun (Bio-Rad, Hercules, CA, USA) with 1100 pounds per square inch rupture disks and a 13 cm distance from the stopping plate to the target [49]. After a recovery period, the explants were grown on MMS media containing 2 mg/L 2,4-D and 1 mg/L bialaphos. After 2-4 weeks of selection, the surviving calli were transferred to regeneration media containing 3 mg/L bialaphos to generate plants [49,50,51]. Nine independent wheat transformation experiments were conducted at staggered intervals. Candidate transgenic plants were re-verified for bialaphos resistance with leaf painting assays [52] and genomic PCR screening for the 4 regions within the CRISPR transformation construct (Table S3). The leaf painting assay utilized Finale^®^ herbicide (BASF Corporation, USA) diluted in water 1:128 (final concentration of the active ingredient glufosinate ammonium was 0.177%). A paintbrush was used to apply a thin layer of the diluted herbicide to a 2 cm long area of a fully expanded young wheat leaf. A wildtype Bobwhite plant of the same age was painted at the same time as a control. The edges of the painted sections were marked with permanent marker, and the leaves were monitored for 3-5 days. The painted sections of transgenic herbicide-resistant leaves remained green, while wildtype sensitive leaves yellowed and/or became necrotic. Thirty-two independent Bobwhite spring wheat transgenic events carrying the CRISPR/Cas transformation construct were generated from these experiments out of 2,945 bombarded immature embryos.

### 4.4. Identification of Events carrying Sal1 Mutations

The transgenic T_0_ wheat plants were screened using a Cleaved Amplified Polymorphic Sequences (CAPS) assay of the 3′ target site (primers in Figure 2C). PCR products were amplified with Taq polymerase (New England Biolabs, USA) and digested with *Xcm*I at 37 °C for 1 h, separated on a 2% agarose gel and visualized with ethidium bromide staining. Sequencing of mutant lines was performed in the T_2_ and T_3_ generations and those mutants were used in subsequent experiments. PCR amplification and cloning was performed to determine the genotypes in these plants.

### 4.5. Quantitative Reverse Transcriptase-PCR

Wheat was grown in a greenhouse with supplemental lighting for 16 h days (maximum 85–90 °F) and 8 h nights (minimum 55 °F). Plants were grown in Sunshine Mix #1 (Sungro Horticulture) in equal-sized pots (9.5 × 9.5 inches) and watered twice daily with fertilizer water (Peter’s 20-20-20 diluted as per instructions) administered by 2 drip lines per pot. During the first three weeks of growth, plants were sprayed with a baking soda solution twice weekly to control powdery mildew (1 tbsp baking soda and 1/2 teaspoon of liquid, non-detergent soap/1 gallon of water). 

For RNA experiments, 6 seeds of each line were planted per pot, then thinned to 5 per pot 7 days after planting. The youngest leaf on each plant was harvested 21 days after planting (approximately Zadoks stage 21-22) and snap frozen in liquid nitrogen and stored at −80 °C until processing. RNA was extracted using the Zymo Quick-RNA Plant miniprep kit (Zymo Research Inc., USA), and the TURBO DNA-free kit was used to remove DNA (Life Technologies, Thermo Fisher, Waltham, MA, USA). Sample RNA concentrations were measured using a DeNovix spectrophotometer (USA); 3 ng/µL RNA was used for each qPCR reaction. Next, 10 µL qRT-PCR reactions were performed with the iTaq Universal SYBR Green One-Step Kit (Bio-Rad, USA) in an Applied Biosystems Quantstudio 3 Realtime PCR System With 96 well 0.1 mL block (Part No. A28566, Thermo Fisher, USA) using the standard conditions recommended in the iTaq manual. Control reactions with no reverse transcriptase were run for each sample using the CJ705892 primers to confirm that there was no DNA contamination [53]. *Sal1* gene expression was normalized relative to the expression of the housekeeping gene CJ705892 [53] using the delta Ct method, and then normalized relative to wildtype Bobwhite. All primers are shown in Figure S6C. *Sal1*-specific primers were designed in Primer3 [47]. Samples were analyzed from three independent biological replicates per experiment from two independent experiments.

### 4.6. Growth and Characterization of Transgenic and Mutant Plants

Wheat was grown in a greenhouse as described in Section 4.5. For growth experiments, 6 seeds of each line were planted per pot, then 12–14 days after germination, the plants were thinned to 4 or 5 plants per pot. For the shared pot experiments, a plastic tag was used to divide the pot into two halves; three seeds from a *Sal1* mutant line were planted on one side, and three Bobwhite seeds were planted on the other, then thinned to two plants per side at 12–14 days. All pots in an experiment were randomly arranged (interspersed with each other) in a 5ft. × 5ft. area on a single greenhouse bench and rearranged weekly to minimize bench position effects. Plant growth (tiller number, leaf number, primary tiller height and development stage) was recorded weekly. Stomatal conductance was measured with an SC-1 Leaf Porometer (Meter Group Inc.) multiple times during two independent drought experiments.

Terminal drought experiments were started 5.5 weeks after planting. At the start of the drought experiments, all pots were hand watered to saturation (maximum soil capacity), then irrigation was halted, and they were not watered again for the duration of the experiment. Two pots were grown per line per experiment, and those placed under the drought treatment was determined by a coin flip. Control plants were watered normally with drip irrigation until their primary stems began senescing, at which point the drip lines were removed and the plants were allowed to naturally senesce. Primary stems were removed intact at harvest; their height was measured with a ruler and internodal diameters were measured with a digital caliper. The total aboveground plant biomass was then harvested into paper bags. Drought treated plants were harvested in the same way once they were fully senesced and dry. Biomass was dried in a drying oven 4–5 days at 30 C, then weighed. Afterward, the seeds were threshed and counted by hand and weighed. Statistical significance was determined by ANOVA. Three independent experiments were performed for the shared pot and drought experiments.

## Figures and Tables

**Table 1:** The *Sal1* gene family in wheat and wheat relatives.

Species	Genomes	Accession Name (Year Sequence Released) [Citation]				*Sal1*			
			5A	5B	5D	4A-1	4A-2	7A	7D
*Aegilops tauschii*	DD	AL8/78, AY61, AY17, XJ02, T093 (2021) [33,34]			**+**				**+**
*Triticum turgidum* subsp. *dicoccoides*	AABB	Zavitan (2019) [35,36]	**+**				**+**	**+**	
*Triticum turgidum* subsp. *durum*	AABB	Svevo (2019) [37]	**+**	**+**		**+**	**+**	**+**	
*Triticum turgidum* subsp. *Durum*	AABB	Kronos (2017)	**+**	**+**		**+**	**+**	**+**	
*Triticum spelta*	AABBDD	PI190962 (2020) [38]	**+**	**+**	**+**	**+**	**+**	**+**	**+**
*Triticum aestivum*	AABBDD	Chinese Spring (2021) [39]	**+**	**+**	**+**	**+**	**+**	**+**	**+**
*Triticum aestivum*	AABBDD	Norin 61 (2020) [38]	**+**	**+**	**+**	**+**	**+**	**+**	**+**
*Triticum aestivum*	AABBDD	Arina*LrFor* (2020) [38]		**+**	**+**	**+**	**+**	**+**	**+**
*Triticum aestivum*	AABBDD	Jagger (2020) [38]		**+**	**+**	**+**	**+**	**+**	**+**
*Triticum aestivum*	AABBDD	Julius (2020) [38]		**+**	**+**	**+**	**+**	**+**	**+**
*Triticum aestivum*	AABBDD	LongReach Lancer (2020) [38]		**+**	**+**	**+**	**+**	**+**	**+**
*Triticum aestivum*	AABBDD	Mace (2020) [38]		**+**	**+**	**+**	**+**	**+**	**+**
*Triticum aestivum*	AABBDD	SY Mattis (2020) [38]		**+**	**+**	**+**	**+**	**+**	**+**
*Triticum aestivum*	AABBDD	Fielder (2021) [40]		**+**	**+**	**+**	**+**	**+**	**+**
*Triticum aestivum*	AABBDD	Bobwhite (this study)		**+**	**+**	**+**	**+**	**+**	**+**
*Triticum aestivum*	AABBDD	CDC Landmark (2020) [38]		**+**	**+**	**+**		**+**	**+**
*Triticum aestivum*	AABBDD	CDC Stanley (2020) [38]		**+**	**+**	**+**		**+**	**+**

**+** = gene present; dark grey boxes = gene absent, light grey boxes indicate the 4A-1 genes with a frameshift mutation resulting in a premature stop codon.

**Table 2:** Genotypes and phenotypes of the wheat lines used in experiments.

Name of Line	Line Description	*Sal1*						Genotype Method	Phenotype	Transgenes Present
		5B	5D	4A-1	4A-2	7A	7D			
**WT**	wildtype Bobwhite	WT	WT	WT	WT	WT	WT	CAPS and sequence	WT	no
**RC**	regeneration control	WT	WT	WT	WT	WT	WT	CAPS	WT	no
**TC1**	transgenic control 1	WT	WT	WT	WT	WT	WT	CAPS	WT	yes
**TC2**	transgenic control 2	WT	WT	WT	WT	WT	WT	CAPS	WT	yes
**PM1**	partial mutant 1	WT	WT	not amplified	WT	mutant *	WT	CAPS and sequence	mutant stems	no
**PM2**	partial mutant 2	WT	mutant	not amplified	not amplified	WT	not amplified	CAPS and sequence	WT	no
**MM**	multiple mutant	mutant	mutant	mutant*	mutant	mutant	mutant	CAPS and sequence	mutant stems	yes
**6KO1**	6 gene knockout 1	mutant	mutant	mutant	mutant	mutant	mutant	CAPS and sequence	mutant stems	yes
**6KO2**	6 gene knockout 2	mutant	mutant	mutant	mutant	mutant	mutant	CAPS and sequence	mutant stems	yes

*: single amino acid deletion or substitution without introducing a frameshift or stop codon.

## Data Availability

The research data are included in the figures and supplementary material associated with the manuscript. The transformation vectors, plant materials and sequenced mutant *Sal1* genotypes are available from the authors upon request.

## Supplementary Materials

The following supporting information can be downloaded at: https://www.mdpi.com/article/10.3390/plants11172259/s1, Figure S1: Expression levels of *Sal1* alleles in different wheat tissues based on publicly available transcriptomic data; Figure S2: Sequences of the targeted regions in the Bobwhite *Sal1* genes. Figure S3: Alignment of the partial *Sal1* protein sequences from *T. aestivum* Bobwhite, *B. distachyon* (Bradi4g40860) and *A. thaliana* (AT5G63980); Figure S4: Diagram and sequence of the *Sal1* CRISPR/Cas9 plasmid vector construct; Figure S5: Major rearrangements, insertions, and deletions seen in 6KO1 and 6KO2 *Sal1* mutants; Figure S6: *Sal1* transcript levels in the 6KO1 and 6KO2 mutants. Figure S7: Growth of well-watered and drought treated *Sal1* mutant lines in single pots; Figure S8: Growth and average seed number of well-watered *Sal1* mutant lines in single and shared pots; Figure S9: Average seed weight of well-watered *Sal1* mutant lines in single and shared pots; Figure S10: Aboveground biomass per plant of drought-treated wildtype and mutant lines in single and shared pots; Table S1: Primers used for genotyping and sequencing *Sal1* alleles. Table S2: Sequences of *Sal1* CRISPR/Cas9 gRNA targets; Table S3: Primers used for confirming transgenes in transgenic wheat lines; Table S4: Detailed description of the independent plant lines used in experiments; Table S5: Detailed annotation of the mutations in the Bobwhite mutant lines.
