# Supplementary material for: CRISPR-Cas9 Gene Editing of the Sal1 Gene Family in Wheat
Source: Plants (Basel). 2022 Aug 30;11(17):2259. doi: 10.3390/plants11172259 (PMC9460255; doi:10.3390/plants11172259)
Supplement: Supplementary file 1 [file plants-11-02259-s001.zip › Mohr Figure S1.pdf]

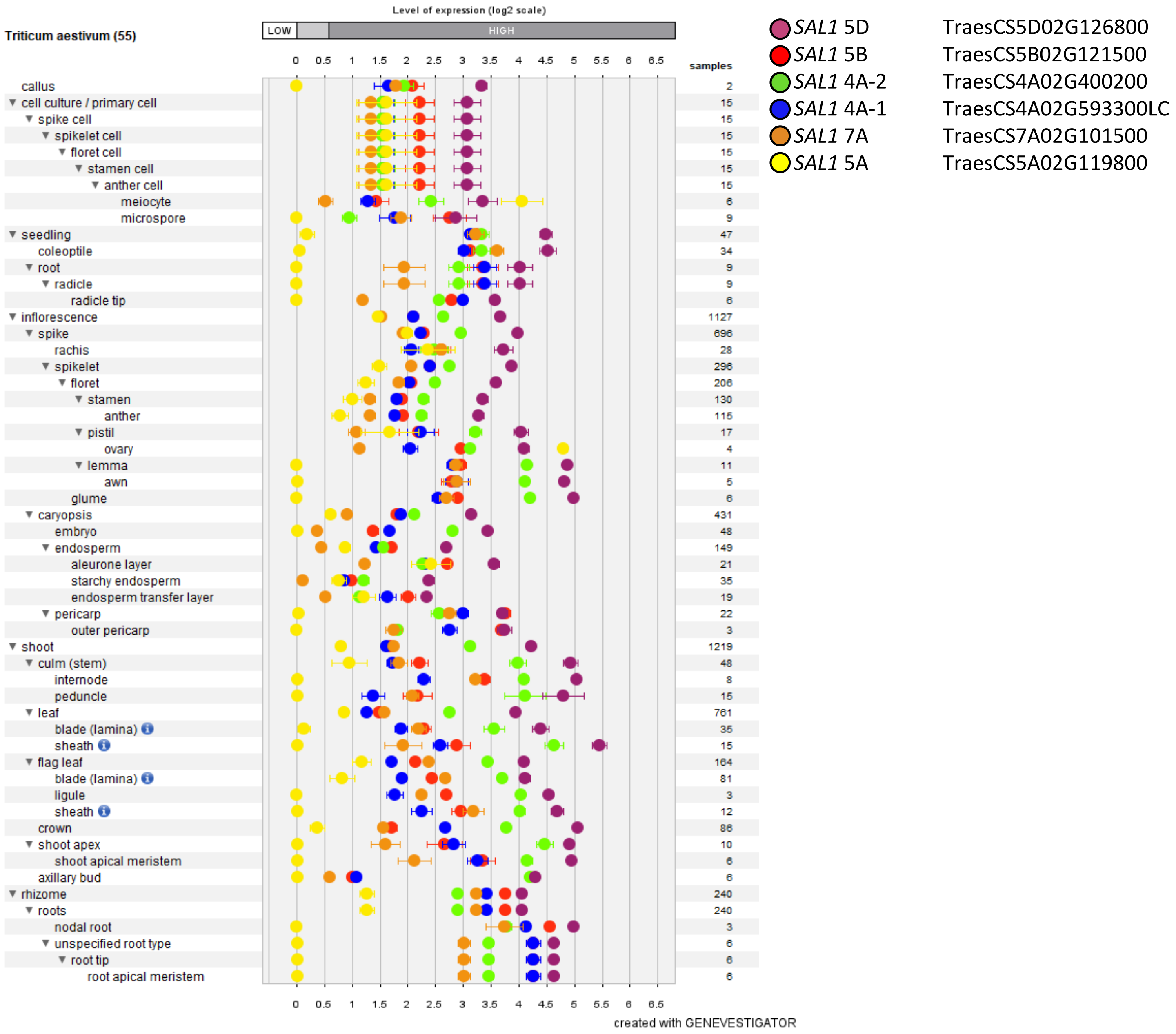

Figure S1: Expression levels of *Sal1* alleles in different wheat tissues based on publicly available transcriptomic data created in Genevestigator (https://genevestigator.com/) April 19, 2022. The 7D homeolog is not shown since it was not annotated in the available datasets.
