# Supplementary material for: CRISPR-Cas9 Gene Editing of the Sal1 Gene Family in Wheat
Source: Plants (Basel). 2022 Aug 30;11(17):2259. doi: 10.3390/plants11172259 (PMC9460255; doi:10.3390/plants11172259)
Supplement: Supplementary file 1 [file plants-11-02259-s001.zip › Mohr Figure S10 revised.pdf]

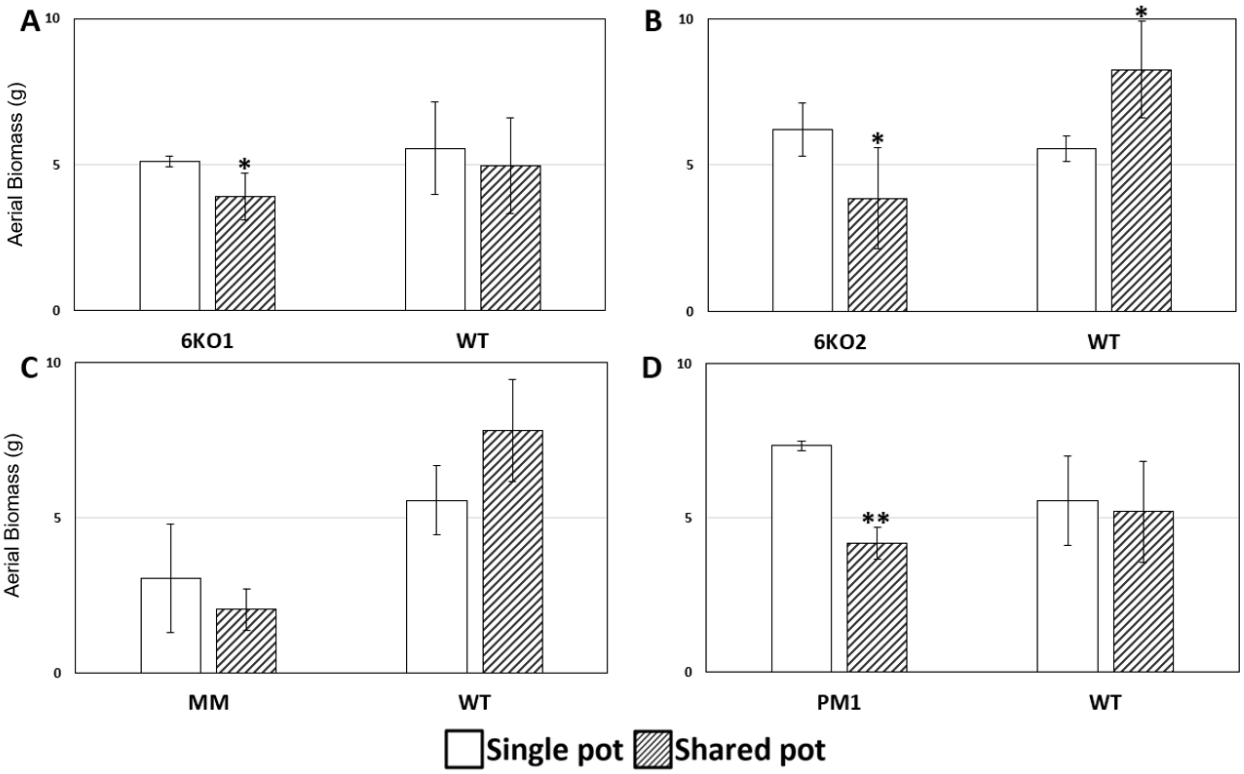

Figure S10. Aboveground biomass per plant (g) of drought-treated wildtype (WT) and mutant lines in single and shared pots. (**A**) 6K01 (**B**) 6KO2 (**C**) MM (**D**) PM1. * = P<0.05; ** = P<0.01, n≥3.
