# Supplementary material for: CRISPR-Cas9 Gene Editing of the Sal1 Gene Family in Wheat
Source: Plants (Basel). 2022 Aug 30;11(17):2259. doi: 10.3390/plants11172259 (PMC9460255; doi:10.3390/plants11172259)
Supplement: Supplementary file 1 [file plants-11-02259-s001.zip › Mohr Figure S2.pdf]

Figure S2:
Sequences of wildtype Bobwhite *Sal1* regions. Exons are in uppercase, introns are in lowercase. Red highlights indicate mutations leading to amino acid changes in Bobwhite relative to Chinese Spring. The yellow highlighted region in *Sal1* 5D indicates the sequence used to interrogate the high-quality wheat genome sequences available at GrainGenes (this region is identical in Bobwhite and Chinese Spring).

>Ta Bobwhite *Sal1* 4A-1
CAAGGCGGATAAGACTCCTGTGACCGTAGCTGATTATGgtacatgctcaccttgcaactgtttacctcca
ggtgcctgtatctgctgtttgatgcttaattagcagtcctcatactcaaaccacaattcaagtcagttcg
atgtttcttgatttaacttacatggagatgttaagatttctgaatggcatggctcagggatgtaaagaac
attcagggtcgtatgatcttcatcagtttacattagcatttggaacttgttcgttctctcaacttcccgt
tcaggtgataatttccttttcatacatgtagGATCCCAAGTGTTGGTAAGTTTTGTGTTAAATATGGAAG
TAACTTCTGGTTCCTTTTCTATGGTGGCTGAGGAGgtaaatcacttgtgtttcggttaaatcgtttctgt
gtataatacataagtattctacacgacctgagtgtttgtatgtacgcagGACTCAGAAGACTTGAGAAAG
GATGGCGCTGAAGAAATTCTTGAGCGCATTACTGATCTTGTAAACAAAACTCTCGCCGAGGATGGTTCAT
ACAACATTTTATTATCTAAGGAAGCTATCCTCTCTGCAATTGATACCGGGAAGTCCGAGGGTGGTCCATC
TGGCCGACATTGGGTTCTAGATCCAATTGATGGGACTAAAGGgtgagctatggtaactccaacctgcagg
tttcacattcttctaatgtgataaaaaagtgcctgtttttccgccaattgcaacatctattgtagtagt
ttctacaactgccttcttgctcgccaaaatcagcgttgctgctttaatttgttgtgagtttttctaatca
ttaattattcaaggtagcaggcaccacactactgccttctttcaaacaactggagttctgacgtttgact
ctaatttctgcatatggaaatgaattcttttactgacgatcttatttaagcagtagagatgccaatatta
ctacggaaatttcgtgaagcagttctatgtaaactatacacggttctcttttcagagatagcttgtgaag
tgctagcaactgatcagttagctggcaagattaagtacctctctttcatgccccatatttgaactaaaca
ttctgtactgattaaccgtgaacactaatagcattgtttgaaaagaaagacattgcagtcattggatgac
acctcatgttgttactatctttgcctataatacattttgatcttggttttgtcttaataacttgattcag
ATTCGTGAGAGGAGGCCAATATGCCATTGCACTGGCACTGCTTGATGAGGGCAGAGTTGTCTTGGGTGTG
TTGGGATGTCCAAATCTTCCTTTTACATCAGTAAGTAACCTCGGTGGTAGCTCATCAGGAGATCAAACCG
GGGCCCTCTTTTCAGCTGCAATCGGTTGTGGTGCTGAAGTACAGTCGTTAGATGGCTCTCCACCACAAAA
Ggttattaccactttcaatctcttttatttgattttaatcagttaaggctgtttgtgcctatgtgagtta
ttttcttttcttgtgattgacttgcttctgggttctggctaactttcattacagATTAGTGTTTGTACCA
TCGACAATCCAGTCAATGCCTCTTTCTTTGAATCCTATGAAGGAGCACACTCGATGCGTGATTTTACTGG
CTCTGTAGCGGAGgtttgatcttgatcactctttgctttggctatatccattgcagtagaaactaattct
gtgtttgagtgctagtagAAACTTGGTGTCCAAGCTCCGCCAGTCAGAATAGATAGCCAAGCAAAATATG
GTGCTCTGGCCCGTGGAGATGGTGCCATTTACTGGCGTTTTCCACACGAGGGTTACAGGAAACGATATGG
GATCATGCAGCCGGCTCAATTGTCGTCACAGgtaagaacatttctctctgtttttttgaaacaacgaactc
aactgaagaatacacatttcttacttccatttttctgatgtttcccagAAGCTGGAGGTGTAGTTAAAGA
TGCCTCAGGAAACGATCTCGATTTCTCgAAAGGCAGATaTCTTGttCGTGACACAGGCATCATTGCcACA
AATAAACAGTTGATGCCATCAGTCCTGAAGTCTGTCCAG

>Ta Bobwhite *Sal1* 4A-2
AAGGCGGATAAGACTCCTGTGACCGTAGCTGATTATGGTACATGCTCACCTTGCAACTGTTTACCTCCAG
GTGCCTGTATCTGTTGTTTGGTGATGCTCAATTAGCAGTCCTCATGCTCAAACCATAATTCAAGTCAGTC
AATGTTTCTTGATTTAACTTACATGGAGATGTTAATATTTCTGAATGACATGGCTCGGGGATGTAAAGAA
CATTCGGGATCGTATGATCTTCGTCAGTTTACATTCACATCTGAAACTTGTTCGTTCTCTCAATTTTTCA
CATTCAGGTGATAATTTTCTTTTCATACATGTAGGATCTCAAGTATTGGTAAGTCTTGTGTTAAATAAGG
AAATAACTTCTGGCTCCTTTTCTATGGTGGCCGAGGAGGTAAATCACTTGTGTTTCGGTTAAATTATTTC
TGTGTATAATACACAAGTATTCTACACGACCTGAGTGTTTGTACGTACGCAGGACTCAGAAGACTTGAGA
AAGGATGGCGCTGAGGAAATTCTGGAGCGCATTACTGATCTTGTAAACAAAACTCTCGCCGAGGATGGTT
CATACAACATTTCATTATCCAAGGAAGCTATCCTCTCTGCAATTGATACCGGGAAGTCCGAGGGAGGTCC
ATCTGGCCGACATTGGGTTCTAGATCCAATTGATGGGACTAAAGGGTGAGCTATGGTAACTCCAACCTGC

AGGTTCCACATTCTGCTAATGTGATAAAAAAAAAGTGCCTGTTTTTTCCGCCAATTGCAACATCTATTGT
AGTAGTTTCTACAACTGCCTTCTTGCTCGCCAAAATCAGCGTTGCTGCTTTAATTTGTTGTGAGTTTTTC
TAATCATTAATTATTCAAGGTAGCAGGCACCACTGCTTTCTTTCAAACAACTGGAGTTCTGACGTTTGAC
TCTAATTTCTGCATACGGAAATGAATTCTTTTACTGACGATCTTATTTAAGCAGTAGAGATGCCAATATT
ACTACGCAAATTTCGTGAAGCAGTTCTATGTAAACTATACACGGTTCTCTTTTCTGAGATAGCTTGTGAA
GTGCTAGCAACTGATCAGTTAGCTGGCAAGATCAAGTACCTCTCTTTCATGCCCCATATTTGAACTAAAC
ATTCTAAACTGATTAACCGTGAACACTAATAGCATTGTTTGAAAAGAAAGACATTGCAGTCATTTGATGA
CAGCTCATGTTGTTACTATCTTTGCCTATAACACATTTTGATCATTTTTTGGTTTTGTCTTAATAACTTG
ATTCAGATTCGTGAGAGGAGGCCAATATGCCATTGCACTGGCACTGCTTGATGAGGGCAGAGTTGTCTTG
GGTGTGCTGGGATGTCCAAATCTTCCTTTTACATCAACAAGTAACCTCAGTGGTAGCTCATCAGGAGATC
AAACCGGGGCCCTCTTTTCAGCGGCAATCGGCTGTGGTGCTGAAGTACAGTCGTTAGATGGCTCTCCACC
ACAAAAGGTTATTACCACTTTCAATCTCTTTTATTTGATTTTAATCAGTTAAGGCTGTTTGTGCCTATGT
GAGTTATTTTCTTTTCTTGTGATTGACTTGCTTCTGGCTAACTTTCATTACAGATTAGTGTTTGTACTAT
TGACAATCCAGCCAATGCCTCGTTCTTTGAATCCTATGAAGGAGCACACACGATGCGTGATTTTACTGGA
TCTGTAGCGGAGGTTTGATCTTGATCACTCTCTGCTTAGGCTATATCCATCGTAGTAGAAACTAATTCTG
TGTTCGAGTGCTACTAGAAACTTGGTGTCCAAGCTCCGCCAGTCAGAATAGATAGCCAAGCAAAATATGG
TGCTCTGGCCCGTGGAGATGGTGCCATTTACTGGCGTTTTCCACACGAGGGTTACAGGGAAACGATATGG
GATCATGCAGCCGGCTCAATTGTCGTCACAGGTAAGAACATTTATCTCTGTTTTTTTTTTCGAAACGATG
AACTCAACTGAAGAATACACATTTCTTACTTCCATTTTTCTGATGTTTCCCAGAAGCTGGAGGTGTAGTT
AAAGATGCCTCAGGAAACGATCTCGATTTCTCGAAAGGCAGATTTCTTGATCGTGACACAGGTATCATTG
CTACAAATAAACAGTTGATGCCATCAGTCCTGAAGTCTGTCCAG

>Ta Bobwhite *Sal1* 5B 5’ target region
tagGATCTCAAGTATTGGTAAGTCTCGTGTTAAATATGGAAGTAACTTCTGGTTCATTTTCTATGGTGGC
CGAAGAGgtaattcacttgtgctgctattaaattattctgtgtttaataatacataagttgttatggcgc
tgctagaaggagggcacgagagggccggccgggggccttttgcccacggccaggcaagagggaagggatt
tccttcttaattcttgcttgattagattgatacatctcctctctttatatagagaggtttacttgactct
caagcaaggcttacttggccccctaagcaaacgacccttatctctaattaaccctaagactaatgggccca
ttaggcccattacgtactcttaacactacaccccacctggacatgcagcttgtcctcgagctgcagccta
accaacttataaccatgactcgacgcaacacaaacctaacacctaaaaacaagccttttacatctcggct
tgttttattattctcaacctgaaatagactaggacgctttattttggacgtgcacgtgtacagccacctg
gatcccatggacaccacctggacaaaaggagtgcatgtgtatggctgttggtctgcaccgcacagggaag
agtggagggcttgcgatggagaatgacgaggaggggtgcgccccccccaaccgccgatgtcgcagactttg
aggtcgttgcccgcggggaaaacagcatgccggccgcccgtgggggaaaccacatgcccaagatccccga
cgcagcggacgagatcgaggtcctttgcgcagcgaagggcaacttggaggagcggcagctgcagaccacg
catctcccgcacacgccgacgcgctaggaggccgcgcgcagcagcctgcagcctcaccgccgccgacacg
tggcgggtagcgatccaagatggaagcagtgacggcgacgacagggacttgatctgctggatgtggacgc
cctgggatggcggcgacgctgatgggaacgaggtcgtcgcactcccgtcgtagggcatcccatactgggc
ggcggagctcaccggcggtggcagctgcagctgcagcggctgctgcggtgtcgtgccgggcagcggcagc
ggttgctgcggcggagcgccgggcgcggcggaggctgctaggagcggcggctgccactgcagccagggtt
gggcggtggtggcgatggatggcagctgcagcggcgcggcggaggccgctaggagcggcggttgccactg
caaccagggttgggcggtggtggcgatggatgacagctgcagcggcgcggcgaaggccgcctggtgcggc
ggctgccatggcagccacggtggcgcagaggcggcgatgggcggcgcagccgggtgcggtccgtaggacc
cggccaagaacaggcggatctcctggaccgcctgggttaggtcccgcagcaccccggacacctcctccca
ggtgaggacagcgggggcggcgcgacggaggatcccgacgcgggcaggagtggggcgacggtcgtggtg
gtcgcgggaggcgacaaggtgaccggcagcggaagagacgggcttggcggcggtgaagacatgatcgaac
cgaagctagctgataccaaattgttatggcgctgctagaaggagggcacgagagggccggccgggggcct
tttgcccacggccaggcaagagggaagggatttccttcttaattcttgcttgattagattgatacatctc
ctctctttatatagagaggtttacttgactctcaagcaaggcttacttggcccctaagcaaacgaccctt
atctctaattaaccctaagactaatgggcccattaggcccattacgtactcttaacataagtattttacg
gttaagtggttgtacatacatagGACTCAGAAGACTTGAGAAAGGATAGTGCTGAAGAAATTCTGGAACA

TATTACTGATCTTGTAAATGAAACTCTCGCTGAGGATGGTTCATACAACATTACTTTATCTAAGGAAGGT
ATCCTCTCTGCAATTGATACTGGGAAGTCTGAAGGAGGTCC

>Ta Bobwhite *Sal1* 5B middle and 3’ target region
agTTTCTTGAGAGGAGGCCAATATGCAATCGCACTGGCACTGCTTGATGAGGGCAAAGTTGTTTTGGGCG
TGTTGGGATGTCCAAATCTTCCTTTGACATCAATAAGCAACCTCAATGGTAGCTCATCAGGAGATCAAAC
AGGGGCCCTCTTTTCAGCTACAATTGGTTGTGGTGCTGAAGTAGAGTCATTAGAGGGCTCTCCACCACAA
AAGgttattaccattttctagctcttttattttgatttgaaatagttaagcccatttctacctatgtgag
atattttgtttttattattgactcgcttctggttaactttcattatagATTAGTGTTTGTACCATTGACA
ATCCAGTGAATGCCTCGTTCTTTGAATCCTATGAAGGAGCACACACAATGCATGATTTAACTGGCTCTAT
AGCGGAGgtttgatcttgactagtctttgcttaggctatatccgttttagtagaaactaattccgtgttt
gagtgctaccagAAACTTGGTGTCCAAGCTCCTCCAGTCAGAATAGATAGCCAAGCAAAATATGGTGCTC
TGGCCCGTGGCGATGGTGCCATTTACTTGCGTTTTCCACACAAGGGTTATAAGGAAAAGATATGGGATCA
TGCAGCTGGCGCAATTGTCGTCACAGgtaatacatttctctttatttttgtaatacatttctctttattt
atttttaaacaaggaactcggctgaaggatacatatttccttactttacgttttcgtgtgttccccagAA
GCTGGAGGTGTAGTAACAGATGCCTCAGGAAACGATCTTGATTTCTCGAAAGGAAGATTTCTTGATGTTG
ACACAGGCATCATTGCCACAAATAAGCAGTTGATGCCATCACTGCTGAAGTCTGTCCAGGA

>Ta Bobwhite *Sal1* 5D
GGTTCATTTTCTATGGTGGCCGAAGAGgtaattcacttgtgttgctattgaattactctgtgtttaatag
tacataagtatttttcggttaagtggttgtacgtacatagGACTCAGAAGACTTGAGAAAGGATAGCGCT
GAAGAAATTCTGGAACATATTACTGATCTTGTAAATGAAACTCTCGCTGAGGATGGTTCATACAACATTA
CTTTATCTAAGGAAGGTATCCTCTCTGCAATTGATACTGGGAAGTCTGAAGGAGGTCCATCTGGCCGACA
TTGGGTTCTGGATCCAATTGATGGGACTAAAGGgtgagctatggtaactgcaacctgcaggtgtcacaat
ctgctaatgcgagaaatgcatttttttcccaccattactctaccatctactctactagttattataagtg
ccttctttctcaccaaaaccagcgttgctgatttgctgtccgtgttcctaatcattagttattcaaggta
gcaggcaccactgccttctttcaaatgacttgaattcctgacctatggttcttaagttcttcatacagaa
tgaattattttaccaattatcttatttaataatttatctgtgtttcttagatctgatttatgcctcgctc
aagtatattggtgcatctcatatgccatcgacaaacatgcgatgatgtgccatttttgcataactggttt
agtgaagcatgctactgtatggatatgttaagcaaaatatcaattctctcttggcatttatgctgatatt
acattgatgtacattttgcccttcctatattaaatagaaaaggtcaataaggtagtgttttatctattac
attgtcttttcaattaagaaaccataagcatgataggcgttgcacataaagcagaagaatttcatgaagt
taaactcatgcaagcaatattacagcaaagggaaaactaaatgtagattcaagggtggattcttgaggtt
agggtttaggtacttgtgcattttgagaaaaatttcaaattaatcatggatgtgcatattgtaagaactt
tttcacctgcgaaaattcatgcaaatatatgataaaaaaataaagaaagaaaagtgttgcaattatacta
ttcaaaaaataagttactttctttttctttttgcgcggggtatgcttgttaataatgttccgtgtttttg
taggtcctcatattatgacatgatgcacatctatgatttgtttttgaaaatttaagctgcagaagtgggt
aaacctaaaatcaagcacacacccttcatgtacaacaaattgataatttgactctacaattgaagggttg
cccttgatcttccatggcacaaacaatttggtagaaatgacattaccatctgacgcaatgagatggaagg
atcatcttacacaatgacaagcagtagaagtgccaatattacttcaaaaaaattgtgaaccagttcttag
taaactgtattctgttcccttatctgagatagctcgtgaagtgctaaaaactgatcagctagctggctag
attgaatgccctttctttcatgtcccatattttagctaaaacattctaacctgatcaatcgtggacagta
cttaccattgtttgaaaagaaagacattccgccatttgatgatggatttttcctgctgaaaaacaatgat
atgcaatgcagcacctcttgttgttactatctttgcctataatacattttgaccatttttgtgttgtttt
aataacttgattcagTTTCTTGAGAGGAGGCCAATATGCAATCGCACTGGCACTGCTTGATGAGGGCAAA
GTTGTTTTGGGCGTGTTGGGATGTCCAAATCTTCCTTTGGCATCAATAAGCAACCTCAATGGTAGCTCAT
GCGGAGATCAAATGGGGGCCCTCTTTTCAGCTACAGTTGGTTGTGGTGCTGAAGTAGAGTCATTAGAGGG
CTCTCCACCACAAAAGgttattaccattttctagctcttttattttgatttgaaatagttaagcctattt
ctacctatgtgagatattttgtttttattattgactcgcttctggttaactttcattatagATTAGTGTT
TGTACCATCGACAATCCAGTGAATGCCTCGTTCTTTGAATCCTATGAAGGAGCACACACAATGCATGATT
TAACTGGCTCTATAGCGGAGgtttgatcttgactagtctttgcttaggctatatccgttttagtagaaac
taataccgtgtttgagtgctaccagAAACTTGGTGTCCAAGCTCCTCCAGTCAGAATAGATAGCCAAGCA

AAATATGGTGCTCTGGCCCGTGGCGATGGTGCCATTTACTTGCGTTTTCCACACAAGGGTTATAAGGAAA
AGATATGGGATCATGCAGCTGGCGCAATTGTCGTCACAGgtaatgcatttctctttattttgtaataca
tttctctttatttatttttaaacaaggaactcggctcaaggatacatatttccttattttcccttttcgt
gtgttccccagAAGCTGGAGGTGTAGTAACAGATGCCTCAGGAAACGATCTCGATTTCTCGAAAGGGAGA
TTTCTTGATGTTGACACAGGCATCATTGCCACAAATAAGCAGTTGATGCCATCACTGCTGAAGTCTGTCC
AGGA

>Ta Bobwhite *Sal1* 7A
CAAGGCGGATAAGACTCCTGTGACCGTAGCTGATTATGgtacatgctcactctgtaactgtttacctcca
ggtgcatgtatctgctgtttgatgctcaattagcagtcctcatactcaaaccataattcgagtcagtcga
tgtttcttgatttaacttacatggagatattaagatttctgaatgacatggctcagtgatgtaaagaaca
ttcagggtcgtatatatgatcttcatcagtttacattcacatttgaaactagttcgttctctcaactccc
cattcaggtgataattttattttcatacatgtagGATCTCAAGTACTGGTAAGTGTTGTGTTAAATATGG
AAGTAACTTCTGGTTCCTTTTCTATGGTGGCCGAGGAGgtaaatcacttgtgttccggttaaatcattta
tgtgtataatacataagtattctacacaacctgagtgtttgtacgtacgcagGACTCAGAAGACTTGAGA
AAGGATGGCGCTGAAGAAATTCTGGAGCGCATTACTGATCTTGTAAACAAAACTCTCGCTGAGGATGGTT
CATACAACCTTTTATTATCTAAGGAAGCTATCCTCTCTGCAATTGATACCGGGAAGTCCGAGGGAGGTCC
ATCTGGCCGACATTGGGTTCTAGATCCAATTGATGGGACTAAAGGgtgagctatggtaactccaacctgc
agatttcacattctgctaatgcgataaaaaaaatgtctggtttttccgccaattgcactatctattgtac
agtagtttctacaactgccctcttgctcaccaaaaccagtgttgctgctttaatttgctgtgcgtttttc
taatcatttattcgaggtagcaggcaccactgccttctttcaaacaactagagttctgacctatgactct
tcatacagaaatgaattcttttaccaacaatcttatttaacaagtacagatgccaatattactacggaaa
tttcctgaaccagctctatgtaaactatccgctgttcttcttttgagatagcttgtgaagcgcgctaaca
actgatcagttagctggcaagattgtatacatctctctcatgccccatattttaactaaacattcgaaac
agattaatcgtgaacactaatagcattgtttgaaaagaaagatattgcagtcatttgatgacacctcatg
ttgttactatctttgcctataatacattttgatcttggttttgtcttaataacttgattcagATTCGTGA
GAGGAGGCCAATATGCCATTGCACTGGCACTGCTTGATGAAGGCAGGGTTGTCTTGGGTGTGTTGGGATG
TCCAAATCTTCCTTTTACATCAGTAAGTAACCTCAGTGGTAGCTCATCAGGAAATCAAACTGGGGCCCTC
TTTTCAGCGGCAATCGGTTGTGGTGCTGAAGTACAGTCGTTAGATGGCTCTCCACCACAAAAGgttatta
tcactttcaatctcttttatttgattttaatcagttaagactgtttgtgcctactctctccgtccggtgc
aaagtgtacatatagagatttttggacaaattatgaagtagagtaaaaaatgcattggaaaggtgcaaac
caccatctctctcatctttaattacccaacctccaatgagctaagtgcatgtagaaattaaggagaccat
gtgtagaatgttattggtcttgattacagtgtgatgagagagaaacatttgtttctattttaaagtgcat
tgggaagatagaagtgcactcttttgtggacaaattttaaaaccaaatgtacactcttcaccgacggagg
gagtatgtgagttattttcttttcttgtgattgacttgcttctggctaactttcattacagATTAGTGTT
TGTACCATCGACAATCCAGCCAATGCCTCGTTCTTTGAATCCTATGAAGGAGCACACTCGATGCGTGATT
TTACTGGCTGTGTAGCGGAGgtttgatcttgattactctttgcttaggctacatccattggagtagaaac
taattctgtgtttgagtgctagtagAAACTTGGTGTCCAAGCTCCTCCAGTCAGAATAGATAGCCAAGCA
AAATATGGTGCCCTGGCCCGTGGGGATGGTGCCATTTACTGGCGTTTTCCACACGAGGGTTACAGGGAAA
CGGTATGGGATCATGCAGCCGGCTCAATTGTCGTCACAGgtaagaacatttctctgtgtgttttttttaa
acaacgaactcaactgaagaatacacatttcttacttccatttttctgatgtttcccagAAGCTGGAGGT
GTAGTTAAAGATGCCTCAGGAAACGATCTCGATTTCTCGAAAGGCAGATATCTTGATCGTGACACGGGCA
TCATTGCTACAAATAAACAGTTGATGCCATCAGTCCTGAAGTCTGTCCAG

>Ta Bobwhite *Sal1* 7D
TGATTATGGTACGTGCTCACTTTGCAACTGTTTACCTCCAGGTGCCTGTATCTGCTGTTTGATGCTCAAT
TAGCAGTCCTCATACTCAAACCATAATTCAAGTCAGTCGATGTTTCTTGATTTAACTTACATGGAGATGT
TAAGATTTCTGAATGACATGGCTCGTGGATGTGAAGAACATTCAGGGTCGTATGATCTTCGTCAGTTTAC
ATTCACATCTGAAACTTGTTTGTTCTCTCAACTTCCCATTCAGGTGATAATTTTCTTTTCATACATGTAG
GGTCTCAAGTATTGGTAAGTCTTGTGTTGAATATGGAAGTAACTTCTGGTTCCTTCTCTATGGTGGCCGA
GGAGGTAAATCACTTGTGTTTCTGTTAAATCATTTCTGTGTATGATACATAAGTATAATACACAACCTGA
GTGTTTGTATGTACGCAGGACTCAGAAGACCTGAGAAAGGATGGCGCTGAAGAAATTCTGGAGCGCATTA

CTGATCTTGTAAACAAAACTCTCGCTGAGGATGGTTCATACAACATTTTATTATCTAAGGAAGCTATCCT
CTCTGCAATTGATACCGGGAAGTCCGAGGGTGGTCCATCTGGCCGACATTGGGTTCTAGATCCAATCGAT
GGGACTAAAGGGTGAGCCGTGGTAACTCCAAGCTGCAGGTTTCACGTTCTGCTAACGCGATAAAAAAATG
CCTGTTTTTTCCGCCAATTTCACCATCTATTGGAGTAGTTTCTACAACTGCCTTCTTGCTCACCAGAAGT
CCAAAACCAGTGTGCTGCTTTAATTTGTTCTGTGTTTTTCTAATCATTAATTATTTAAGGTACCACGCAC
CACTGCCTTCTTTCAAACAACTAGAGTTCTGACCTATGACTCTGCATACAGAAATGAATTCTTTTACCAA
CAATCTTATTTAACAAGTAGAGATGCCAATATTACTACAGAAATTTTGTGAAGCAGTTCTATGTAAACTA
TACACGGTTCTCTTTTCTGAGATAGCTTGTGAAGTGCTAACAACTGATCAGTTAGCTGGCAAGATTAAGT
ACCTCTCTTTCATGCCCCATATTTTAACTAAACGTTCTAAACTGATTAATCGTGAACACTAATAGCATTT
GTTTGAAAAGAAAGACATTGCAGTCATTTGATGACAGCTCATGTTGTTACTATCTCTGCCTATAATACAT
TTTGATCATTTTTTGGTTTTGTCTTAATAACTTGATTCAGATTCGTGAGAGGAGGCCAATATGCCATTGC
ACTGGCACTGCTTGATGAGGGCAGAGTTGTCTTGGGTGTGCTGGGATGTCCAAATCTTCCTTTTACATCA
GTAAGTAACCTCAGTGGTAGCTCATCAGGAGATCAAACTGGGGCCCTCTTTTCAGCTGCAATCGGTTGTG
GTGCTGAAGTACAGTCGTTAGATGGCTCTCCACCACAAAAGGTTATTACCACTTTCAATCTCTTTTATTT
GATTTTAATCAGTGATGGCTGTTTGTGCCTATGTGAGTTATTTTCTTTTCTTGTGATTGACTTGCTTCTG
GCTAACTTTCATTACAGATCAGTGTTTGTACCATCGACAATCCAGCCAATGCCTCGTTCTTTGAATCCTA
TGAAGGAGCACACTCAATGCGTGATTTTACTGGCTCTGTAGCGGAGGTTTGATCTTGATCACTCTTTGAT
TAGGCTCTATCCATTCGAGTAGAAACTAATTCTGTGTTTGAGTGCTAGTAGAAACTTGGTGTCCAAGCTC
CTCCAGTCAGAATAGATAGCCAAGCAAAATATGGCGCTCTGGCCCGTGGAGATGGTGCCATTTACTGGCG
TTTTCCACACGAGGGTTACAGGGAAACGATATGGGATCATGCAGCCGGGTCAATTGTCGTCACAGGTAAG
AACATTGCTCTCTGTTTTTGAAACAATGAACTCAACTGAAG
