# Supplementary material for: CRISPR-Cas9 Gene Editing of the Sal1 Gene Family in Wheat
Source: Plants (Basel). 2022 Aug 30;11(17):2259. doi: 10.3390/plants11172259 (PMC9460255; doi:10.3390/plants11172259)
Supplement: Supplementary file 1 [file plants-11-02259-s001.zip › Mohr Figure S3.pdf]

```
CLUSTAL O(1.2.4) multiple sequence alignment

A_thaliana_SAL1        EPFSLVAEEDSGDLRKDGSQDTLERITKLVNDTLATEESFNGSTLSTDDLLRAIDCGTSE   60
B_distachyon_SAL1      GSFSMVAEEDSEDLRKEGAEEILEHITDLVNETLAEDGSFNI-SLSQEGILSAIDSGKSE   59
BW_Sal1_5D             GSFSMVAEEDSEDLRKDSAEEILEHITDLVNETLAEDGSYNI-TLSKEGILSAIDTGKSE   59
BW_Sal1_5B             GSFSMVAEEDSEDLRKDSAEEILEHITDLVNETLAEDGSYNI-TLSKEGILSAIDTGKSE   59
BW_Sal1_7A             GSFSMVAEEDSEDLRKDGAEEILERITDLVNKTLAEDGSYNL-LLSKEAILSAIDTGKSE   59
BW_Sal1_7D             GSFSMVAEEDSEDLRKDGAEEILERITDLVNKTLAEDGSYNI-LLSKEAILSAIDTGKSE   59
BW_Sal1_4A-1           GSFSMVAEEDSEDLRKDGAEEILERITDLVNKTLAEDGSYNI-LLSKEAILSAIDTGKSE   59
BW_Sal1_4A-2           GSFSMVAEEDSEDLRKDGAEEILERITDLVNKTLAEDGSYNI-SLSKEAILSAIDTGKSE   59
                         **:****** ****:.:::  **:**.***.*** : *:*    ** : :* *** *.**

A_thaliana_SAL1        GGPNGRHWVLDPIDGTKGFLRGDQYAVALGLLEEGKVVLGVLACPNLPLASIAGNNKNKS   120
B_distachyon_SAL1      GGPSGRHWVLDPIDGTKGFVRGGQYAIALALLDEGKVVLGVLGCPNLPLTSICNLN-GNS   118
BW_Sal1_5D             GGPSGRHWVLDPIDGTKGFLRGGQYAIALALLDEGKVVLGVLGCPNLPLASISNLN-GSS   118
BW_Sal1_5B             G[redacted]GFLRGGQYAIALALLDEGKVVLGVLGCPNLPLTSISNLN-GSS   102
BW_Sal1_7A             GGPSGRHWVLDPIDGTKGFVRGGQYAIALALLDEGRVVLGVLGCPNLPFTSVSNLS-GSS   118
BW_Sal1_7D             GGPSGRHWVLDPIDGTKGFVRGGQYAIALALLDEGRVVLGVLGCPNLPFTSVSNLS-GSS   118
BW_Sal1_4A-1           GGPSGRHWVLDPIDGTKGFVRGGQYAIALALLDEGRVVLGVLGCPNLPFTSVSNLG-GSS   118
BW_Sal1_4A-2           GGPSGRHWVLDPIDGTKGFVRGGQYAIALALLDEGRVVLGVLGCPNLPFTSTSNLS-GSS   118
                       *                 **:**.***:**.**:**:******.*****::*  ..  .  ..*

A_thaliana_SAL1        SSDEIGCLFFATIGSGTYMQLLDSKSSPVKVQVSSVENPEEASFFESFEGAHSLHDLSSS   180
B_distachyon_SAL1      SGDQTGVLFSATIGCGAEVQSLDG-SPPQKISVCSIDNPVNASFFESYEGAHNMRDVTGS   177
BW_Sal1_5D             CGDQMGALFSATVGCGAEVESLEG-SPPQKISVCTIDNPVNASFFESYEGAHTMHDLTGS   177
BW_Sal1_5B             SGDQTGALFSATIGCGAEVESLEG-SPPQKISVCTIDNPVNASFFESYEGAHTMHDLTGS   161
BW_Sal1_7A             SGNQTGALFSAAIGCGAEVQSLDG-SPPQKISVCTIDNPANASFFESYEGAHSMRDFTGC   177
BW_Sal1_7D             SGDQTGALFSAAIGCGAEVQSLDG-SPPQKISVCTIDNPANASFFESYEGAHSMRDFTGS   177
BW_Sal1_4A-1           SGDQTGALFSAAIGCGAEVQSLDG-SPPQKISVCTIDNPVNASFFESYEGAHSMRDFTGS   177
BW_Sal1_4A-2           SGDQTGALFSAAIGCGAEVQSLDG-SPPQKISVCTIDNPANASFFESYEGAHTMRDFTGS   177
                       ..:: * ** *::*.*: :: *:. * * *:.*.:::** :******:****.::*.:..

A_thaliana_SAL1        IANKLGVKAPPVRIDSQAKYGALSRGDGAIYLRFPHKGYREKIWDHVAGAIVVTEAGGIV   240
B_distachyon_SAL1      IAEKLGVQAPPVRIDSQAKYGALARGDGAIYLRFPHKGYKEKIWDHAAGSIVVTEAGGIV   237
BW_Sal1_5D             IAEKLGVQAPPVRIDSQAKYGALARGDGAIYLRFPHKGYKEKIWDHAAGAIVVTEAGGVV   237
BW_Sal1_5B             IAEKLGVQAPPVRIDSQAKYGALARGDGAIYLRFPHKGYKEKIWDHAAGAIVVTEAGGVV   221
BW_Sal1_7A             VAEKLGVQAPPVRIDSQAKYGALARGDGAIYWRFPHEGYRETVWDHAAGSIVVTEAGGVV   237
BW_Sal1_7D             VAEKLGVQAPPVRIDSQAKYGALARGDGAIYWRFPHEGYRETIWDHAAGSIVVTEAGGVV   237
BW_Sal1_4A-1           VAEKLGVQAPPVRIDSQAKYGALARGDGAIYWRFPHEGYRKRYGIMQPAQLSSQKLEV*-   235
BW_Sal1_4A-2           VAEKLGVQAPPVRIDSQAKYGALARGDGAIYWRFPHEGYRETIWDHAAGSIVVTEAGGVV   237
                       :*:****:***************:******* ****:**::        . :   :
```

**Figure S3:** Alignment of the partial Sal1 protein sequences from *T. aestivum* cv. Bobwhite, *B. distachyon* (Bradi4g40860) and *A. thaliana* (AT5G63980). The proteins were trimmed to remove the regions not sequenced in Bobwhite (outside of the regions targeted for CRISPR editing). There is a gap at residue 60 in the 5B gene that was not able to be amplified and sequenced (black box). Highlighted areas show the conserved regions targeted by the 5′ (maroon), middle (green) and 3′ (teal) gRNAs. Cysteine residues conserved in the grasses are shown in red text. Amino acids highlighted in yellow are unique to a portion of the wheat homeologs. The different amino acids caused by the single base pair deletion in 4A-1 are highlighted in gray.
