# Supplementary material for: CRISPR-Cas9 Gene Editing of the Sal1 Gene Family in Wheat
Source: Plants (Basel). 2022 Aug 30;11(17):2259. doi: 10.3390/plants11172259 (PMC9460255; doi:10.3390/plants11172259)
Supplement: Supplementary file 1 [file plants-11-02259-s001.zip › Mohr Figure S4.pdf]

Figure S4. CRISPR/Cas9 construct map and plasmid sequence.

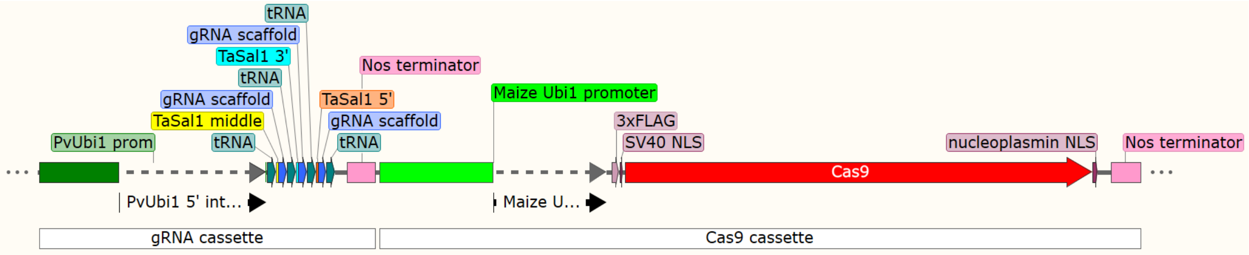

Sequence of the CRISPR plasmid vector is shown below. Regions are marked in the sequence as follows: **PvUbi promoter**, *PvUbi1 5' intron*, tRNA, gRNA scaffold, **guide RNA sequence,** Nos terminator, Maize Ubi1 promoter, *Maize Ubi1 5' intron*, 3X FLAG, SV40 NLS, Cas9, nucleoplasmin NLS, plasmid vector backbone.

>Wheat CRISPR/Cas9 plasmid sequence

CCACTGGAGAGGGGCACACACGTCAGTGTTTGGTTTCCACTAGCACGAGTAGCGCAATCAGAAAATTTTCAATGCAT
GAAGTACTAAACGAAGTTTATTTAGAAATTTTTTTAAGAAATGAGTGTAATTTTTTGCGACGAATTTAATGACAATA
ATTAATCGATGATTGCCTACAGTAATGCTACAGTAACCAACCTCTAATCATGCGTCGAATGCGTCATTAGATTCGTC
TCGCAAAATAGCACAAGAATTATGAAATTAATTTTACAAACTATTTTTATTTAATACTAATAATTAACTGTCAAAGT
TTGTGCTACTCGCAAGAGTAGCGCGAACCAAACACGGCCTGGAGGAGCACGGTAACGGCGTCtACAAACTAACGGCC
ACCACCCGCCAACGCAAAGGAGACGGATGAGAGTTGACTTCTTGACGGTTCTCCACCCCTCTGTCTCTCTGTCACTG
GGCCCTGGGTCCCCCTCTCGAAAGTTCCTCTGGCCGAAATTGCGCGGCGGAGACGAGGCGGGCGGAACCGTCACGGC
AGAGGATTCCTTCCCCACCCTGCCTGGCCCGGCCATATATAAACAGCCACCGCCCCTCCCCGTTCCCCATCGCGTCT
CGTCTCGTGTTGTTCCCAGAACACAACCAAAATCCAAATCCTCCTCCTCCTCCCGAGCCTCGTCGATCCCTCACCCG
CTTCAAGgtacggcgatcctcctctcccttctcccctcgatcgattatgcgtgttccgtttccgtttccgatcgagc
gaatcgatggttaggacctatggggacccattgggtgtcgtgtggtggtctggtttgatccgcgatatttctccgt
tcgtagtgtagatctgatcgaatccctggtgaaatcgttgatcgtgctattcgtgtgagggttcttaggtttggagt
tgtggaggtagttctgatcggtttgtaggtgagattttccccatgattttgcttggctcgtttgtcttggttagatt
agatctgcccgcattttgttcgatatttctgatgcagatatgatgaataatttcgtccttgtatcccgcgtccgtat
gtgtattaagtttgcaggtgctagttaggttttcctactgatttgtcttatccattctgtttagcttgcaaggttt
ggtaatggtccggcatgtttgtctctatagattagagtagaataagattatctcaacaagctgttggcttatcaatt
ttggatctgcatgtgtttcgcatctatatctttgcaattaagatggtagatggacatatgctcctgttgagttgatg
ttgtacctttacctgaggtctgaggaacatgcatcctcctgctactttgtgcttatacagatcatcaagattatgc
agctaatattcgatcagtttctagtatctacatggtaaacttgcatgcacttgctacttatttttgatatacttgga
tgataacatatgctgctggttgattcctacctacatgatgaacattttacaggccattagtgtctgtctgtatgtgt
tgttcctgtttgcttcagtctatttctgtttcattcctagtttattggttctctgctagatacttaccctgctgggc
ttagttatcatcttatctcgaatgcattttcatgtttatagatgaatatactcagataggtgtagatgtatgcta
ctgtttctctacgttgctgtaggtttacctgtggcaactgcatactcctgttgcttcgctagatatgtatgtgctt
atatagattaagatatgtgtgatggttctttagtatatctgatgatcatgtatgctcttttaacttcttgctacact
tggtaacatgctgtgatgctgtttgttgattctgtagcactaccaatgatgaccttatctctctttgtatatgatgt
ttctgtttgtttgaggcttgtgttactgctagttacttaccctgttgcctggctaatcttctgcagggatccCcatg
gaacaaagcaccagtggtctagtggtagaatagtaccctgccacggtacagacccgggttcgattcccggctggtgc
a**GCACTGGCACTGCTTGATGA**gttttagagctagaaatagcaagttaaaataaggctagtccgttatcaacttgaaa
aagtggcaccgagtcggtgcaacaaagcaccagtggtctagtggtagaatagtaccctgccacggtacagacccggg
ttcgattcccggctggtgca**ATAGATAGCCAAGCAAAATA**gttttagagctagaaatagcaagttaaaataaggcta
gtccgttatcaacttgaaaaagtggcaccgagtcggtgcaacaaagcaccagtggtctagtggtagaatagtaccct
gccacggtacagacccgggttcgattcccggctggtgca**GGACTCAGAAGACTTGAGAA**gttttagagctagaaata
gcaagttaaaataaggctagtccgttatcaacttgaaaaagtggcaccgagtcggtgcaacaaagcaccagtggtct
agtggtagaatagtaccctgccacggtacagacccgggttcgattcccggctggtgcaGCTAGCCATCACCATCACC
ATCACGTGTGACCTGCAGGTCTAGTGGTACCGCCCCGTCCGGTCCTGCCCGTCACCGAGATCTGATCCGTCGACCTG
CAGATCGTTCAAACATTTGGCAATAAAGTTTCTTAAGATTGAATCCTGTTGCCGGTCTTGCGATGATTATCATATAA
TTTCTGTTGAATTACGTTAAGCATGTAATAATTAACATGTAATGCATGACGTTATTTATGAGATGGGTTTTTATGAT

TAGAGTCCCGCAATTATACATTTAATACGCGATAGAAAACAAAATATAGCGCGCAAACTAGGATAAATTATCGCGCG
CGGTGTCATCTATGTTACTAGATCCGATGATAAGCTGTCAAACATGAGAATTAAACaaaCCCGGctgcagtgcagcg
tgacccggtcgtgcccctctctagagataatgagcattgcatgtctaagttataaaaaattaccacatattttttt
gtcacacttgtttgaagtgcagtttatctatctttatacatatatttaaactttactctacgaataatataatctat
agtactacaataatatcagtgttttagagaatcatataaatgaacagttagacatggtctaaaggacaattgagtat
tttgacaacaggactctacagttttatctttttagtgtgcatgtgttctcctttttttttgcaaatagcttcaccta
tataatacttcatccattttattagtacatccatttagggtttagggttaatggtttttatagactaattttttag
tacatctattttattctattttagcctctaaattaagaaaactaaaactctattttagtttttttatttaataattt
agatataaaatagaataaaataaagtgactaaaaattaaacaaataccctttaagaaattaaaaaaactaaggaaac
atttttcttgtttcgagtagataatgccagcctgttaaacgccgtcgacgagtctaacggacaccaaccagcgaacc
agcagcgtcgcgtcgggccaagcgaagcagacggcacggcatctctgtcgctgcctctggacccctctcgagagttc
cgctccaccgttggacttgctccgctgtcggcatccagaaattgcgtggcggagcggcagacgtgagccggcacggc
aggcggcctcctcctcctctcacggcaccggcagctacgggggattcctttcccaccgctccttcgctttcccttcc
tcgcccgccgtaataaatagacacccctccacaccctctttccccaacctcgtgttgttcggagcgcacacacaca
caaccagatctcccccaaatccacccgtcggcacctccgcttcaaggtacgccgctcgtcctcccccccccccccctc
tctaccttctctagatcggcgttccggtccatggttagggcccggtagttctacttctgttcatgtttgtgttagat
ccgtgtttgtgttagatccgtgctgctagcgttcgtacacggatgcgacctgtacgtcagacacgttctgattgcta
acttgccagtgtttctctttggggaatcctgggatggctctagccgttccgcagacgggatcgatttcatgatttttt
tttgtttcgttgcatagggtttggtttgcccttttcctttatttcaatatatgccgtgcacttgtttgtcgggtcat
cttttcatgcttttttttgtcttggttgtgatgatgtggtctggttgggcggtcgttctagatcggagtagaaatct
gtttcaaactacctggtggatttattaattttggatctgtatgtgtgtgccatacatattcatagttacgaattgaa
gatgatggatggaaatatcgatctaggataggtatacatgttgatgcgggttttactgatgcatatacagagatgct
ttttgttcgcttggttgtgatgatgtggtgtggttgggcggtcgttcattcgttctagatcggagtagaatactgtt
tcaaactacctggtgtatttattaattttggaactgtatgtgtgtgtcatacatcttcatagttacgagtttaagat
ggatggaaatatcgatctaggataggtatacatgttgatgtgggttttactgatgcatatacatgatggcatatgca
gcatctattcatatgctctaaccttgagtacctatctattataataaacaagtatgttttataattattttgatctt
gatatacttggatgatggcatatgcagcagctatatgtggatttttttagccctgccttcatacgctatttatttgc
ttggtactgtttcttttgtcgatgctcaccctgttgtttggtgttacttctgcagTTcgaaggagatagaaccaatt
ctctaaggaaatacttaaccATGgactataaggaccacgacggagactacaaggatcatgatattgattacaaagac
gatgacgataagatggccccaaagaagaagcggaaggtcggtatccacggagtcccagcagccgacaagaagtacag
catcggcctggacatcggcaccaactctgtgggctgggccgtgatcaccgacgagtacaaggtgcccagcaagaaat
tcaaggtgctgggcaacaccgaccggcacagcatcaagaagaacctgatcggagccctgctgttcgacagcggcgaa
acagccgaggccacccggctgaagagaaccgccagaagaagatacaccagacggaagaaccggatctgctatctgca
agagatcttcagcaacgagatggccaaggtggacgacagcttcttccacagactggaagagtccttcctggtggaag
aggataagaagcacgagcggcaccccatcttcggcaacatcgtggacgaggtggcctaccacgagaagtaccccacc
atctaccacctgagaaagaaactggtggacagcaccgacaaggccgacctgcggctgatctatctggccctggccca
catgatcaagttccggggccacttcctgatcgagggcgacctgaaccccgacaacagcgacgtggacaagctgttca
tccagctggtgcagacctacaaccagctgttcgaggaaaaccccatcaacgccagcggcgtggacgccaaggccatc
ctgtctgccagactgagcaagagcagacggctggaaaatctgatcgcccagctgcccggcgagaagaagaatggcct
gttcggaaacctgattgccctgagcctgggcctgacccccaacttcaagagcaacttcgacctggccgaggatgcca
aactgcagctgagcaaggacacctacgacgacgacctggacaacctgctggcccagatcggcgaccagtacgccgac
ctgtttctggccgccaagaacctgtccgacgccatcctgctgagcgacatcctgagagtgaacaccgagatcaccaa
ggcccccctgagcgcctctatgatcaagagatacgacgagcaccaccaggacctgaccctgctgaaagctctcgtgc
ggcagcagctgcctgagaagtacaaagagattttcttcgaccagagcaagaacggctacgccggctacattgacggc
ggagccagccaggaagagttctacaagttcatcaagcccatcctggaaaagatggacggcaccgaggaactgctcgt
gaagctgaacagagaggacctgctgcggaagcagcggaccttcgacaacggcagcatcccccaccagatccacctgg
gagagctgcacgccattctgcggcggcaggaagatttttacccattcctgaaggacaaccgggaaaagatcgagaag
atcctgaccttccgcatcccctactacgtgggccctctggccaggggaaacagcagattcgcctggatgaccagaaa
gagcgaggaaaccatcaccccctggaacttcgaggaagtggtggacaagggcgcttccgcccagagcttcatcgagc
ggatgaccaacttcgataagaacctgcccaacgagaaggtgctgcccaagcacagcctgctgtacgagtacttcacc
gtgtataacgagctgaccaaagtgaaatacgtgaccgagggaatgagaaagcccgccttcctgagcggcgagcagaa
aaaggccatcgtggacctgctgttcaagaccaaccggaaagtgaccgtgaagcagctgaaagaggactacttcaaga
aaatcgagtgcttcgactccgtggaaatctccggcgtggaagatcggttcaacgcctccctgggcacataccacgat
ctgctgaaaattatcaaggacaaggacttcctggacaatgaggaaaacgaggacattctggaagatatcgtgctgac
cctgacactgtttgaggacagagagatgatcgaggaacggctgaaaacctatgcccacctgttcgacgacaaagtga
tgaagcagctgaagcggcggagatacaccggctggggcaggctgagccggaagctgatcaacggcatccgggacaag
cagtccggcaagacaatcctggatttcctgaagtccgacggcttcgccaacagaaacttcatgcagctgatccacga

cgacagcctgacctttaaagaggacatccagaaagcccaggtgtccggccagggcgatagcctgcacgagcacattg
ccaatctggccggcagccccgccattaagaagggcatcctgcagacagtgaaggtggtggacgagctcgtgaaagtg
atgggccggcacaagcccgagaacatcgtgatcgaaatggccagagagaaccagaccacccagaagggacagaagaa
cagccgcgagagaatgaagcggatcgaagagggcatcaaagagctgggcagccagatcctgaaagaacaccccgtgg
aaaacacccagctgcagaacgagaagctgtacctgtactacctgcagaatgggcgggatatgtacgtggaccaggaa
ctggacatcaaccggctgtccgactacgatgtggaccatatcgtgcctcagagctttctgaaggacgactccatcga
caacaaggtgctgaccagaagcgacaagaaccggggcaagagcgacaacgtgccctccgaagaggtcgtgaagaaga
tgaagaactactggcggcagctgctgaacgccaagctgattacccagagaaagttcgacaatctgaccaaggccgag
agaggcggcctgagcgaactggataaggccggcttcatcaagagacagctggtggaaacccggcagatcacaaagca
cgtggcacagatcctggactcccggatgaacactaagtacgacgagaatgacaagctgatccgggaagtgaaagtga
tcaccctgaagtccaagctggtgtccgatttccggaaggatttccagttttacaaagtgcgcgagatcaacaactac
caccacgcccacgacgcctacctgaacgccgtcgtgggaaccgccctgatcaaaaagtaccctaagctggaaagcga
gttcgtgtacggcgactacaaggtgtacgacgtgcggaagatgatcgccaagagcgagcaggaaatcggcaaggcta
ccgccaagtacttcttctacagcaacatcatgaactttttcaagaccgagattaccctggccaacggcgagatccgg
aagcggcctctgatcgagacaaacggcgaaaccggggagatcgtgtgggataagggccgggattttgccaccgtgcg
gaaagtgctgagcatgccccaagtgaatatcgtgaaaaagaccgaggtgcagacaggcggcttcagcaaagagtcta
tcctgcccaagaggaacagcgataagctgatcgccagaaagaaggactgggaccctaagaagtacggcggcttcgac
agccccaccgtggcctattctgtgctggtggtggccaaagtggaaaagggcaagtccaagaaactgaagagtgtgaa
agagctgctggggatcaccatcatggaaagaagcagcttcgagaagaatcccatcgactttctggaagccaagggct
acaaagaagtgaaaaaggacctgatcatcaagctgcctaagtactccctgttcgagctggaaaacggccggaagaga
atgctggcctctgccggcgaactgcagaagggaaacgaactggccctgccctccaaatatgtgaacttcctgtacct
ggccagccactatgagaagctgaagggctcccccgaggataatgagcagaaacagctgtttgtggaacagcacaagc
actacctggacgagatcatcgagcagatcagcgagttctccaagagagtgatcctggccgacgctaatctggacaaa
gtgctgtccgcctacaacaagcaccgggataagcccatcagagagcaggccgagaatatcatccacctgtttaccct
gaccaatctgggagcccctgccgccttcaagtactttgacaccaccatcgaccggaagaggtacaccagcaccaaag
aggtgctggacgccaccctgatccaccagagcatcaccggcctgtacgagacacggatcgacctgtctcagctggga
ggcgacaaaaggccggcggccacgaaaaaggccggccaggcaaaaaagaaaaagTAAgaattcgcggccgcactcga
gatatctagacccagctttcttgtacaaagtggttgataacagcgactacaaggatgacgatgacaaggcttagagc
tcgaatttccccgatcgttcaaacatttggcaataaagtttcttaagattgaatcctgttgccggtcttgcgatgat
tatcatataatttctgttgaattacgttaagcatgtaataattaacatgtaatgcatgacgttatttatgagatggg
tttttatgattagagtcccgcaattatacatttaatacgcgatagaaaacaaaatatagcgcgcaaactaggataaa
ttatcgcgcgcggtgtcatctatgttactagatcgggaattcactggccgtcgttttacactggccgtcgttttaca
acgtcgtgactgggaaaaccctggcgttacccaacttaatcgccttgcagcacatccccctttcgccagctggcgta
atagcgaagaggcccgcaccgatcgcccttcccaacagttgcgcagcctgaatggcgaatgctagagcagcttgagc
ttggatcagattgtcgtttcccgccttcagtttgcatCCTAGTGGTACCGCCCCGTCCGGTCCTGCCCGTCACCGAG
ATCTGATCCGTCGACCTGCAGATCGTTCAAACATTTGGCAATAAAGTTTCTTAAGATTGAATCCTGTTGCCGGTCTT
GCGATGATTATCATATAATTTCTGTTGAATTACGTTAAGCATGTAATAATTAACATGTAATGCATGACGTTATTTAT
GAGATGGGTTTTTATGATTAGAGTCCCGCAATTATACATTTAATACGCGATAGAAAACAAAATATAGCGCGCAAACT
AGGATAAATTATCGCGCGCGGTGTCATCTATGTTACTAGATCCGATGATAAGCTGTCAAACATGAGAATTATAACTT
CGTATAGCATACATTATACGAAGTTATGAGCTCGAATTCGTAATCATGGTCATAGCTGTTTCCTGTGTGAAATTGTT
ATCCGCTCACAATTCCACACAACATACGAGCCGGAAGCATAAAGTGTAAAGCCTGGGGTGCCTAATGAGTGAGCTAA
CTCACATTAATTGCGTTGCGCTCACTGCCCGCTTTCCAGTCGGGAAACCTGTCGTGCCAGCTGCATTAATGAATCGG
CCAACGCGCGGGGAGAGGCGGTTTGCGTATTGGGCGCTCTTCCGCTTCCTCGCTCACTGACTCGCTGCGCTCGGTCG
TTCGGCTGCGGCGAGCGGTATCAGCTCACTCAAAGGCGGTAATACGGTTATCCACAGAATCAGGGGATAACGCAGGA
AAGAACATGTGAGCAAAAGGCCAGCAAAAGGCCAGGAACCGTAAAAAGGCCGCGTTGCTGGCGTTTTTCCATAGGCT
CCGCCCCCCTGACGAGCATCACAAAAATCGACGCTCAAGTCAGAGGTGGCGAAACCCGACAGGACTATAAAGATACC
AGGCGTTTCCCCCTGGAAGCTCCCTCGTGCGCTCTCCTGTTCCGACCCTGCCGCTTACCGGATACCTGTCCGCCTTT
CTCCCTTCGGGAAGCGTGGCGCTTTCTCATAGCTCACGCTGTAGGTATCTCAGTTCGGTGTAGGTCGTTCGCTCCAA
GCTGGGCTGTGTGCACGAACCCCCCGTTCAGCCCGACCGCTGCGCCTTATCCGGTAACTATCGTCTTGAGTCCAACC
CGGTAAGACACGACTTATCGCCACTGGCAGCAGCCACTGGTAACAGGATTAGCAGAGCGAGGTATGTAGGCGGTGCT
ACAGAGTTCTTGAAGTGGTGGCCTAACTACGGCTACACTAGAAGGACAGTATTTGGTATCTGCGCTCTGCTGAAGCC
AGTTACCTTCGGAAAAAGAGTTGGTAGCTCTTGATCCGGCAAACAAACCACCGCTGGTAGCGGTGGTTTTTTTGTTT
GCAAGCAGCAGATTACGCGCAGAAAAAAAGGATCTCAAGAAGATCCTTTGATCTTTTCTACGGGGTCTGACGCTCAG
TGGAACGAAAACTCACGTTAAGGGATTTTGGTCATGAGATTATCAAAAAGGATCTTCACCTAGATCCTTTTAAATTA
AAAATGAAGTTTTAAATCAATCTAAAGTATATATGAGTAAACTTGGTCTGACAGTTACCAATGCTTAATCAGTGAGG
CACCTATCTCAGCGATCTGTCTATTTCGTTCATCCATAGTTGCCTGACTCCCCGTCGTGTAGATAACTACGATACGG
GAGGGCTTACCATCTGGCCCCAGTGCTGCAATGATACCGCGAGACCCACGCTCACCGGCTCCAGATTTATCAGCAAT

AAACCAGCCAGCCGGAAGGGCCGAGCGCAGAAGTGGTCCTGCAACTTTATCCGCCTCCATCCAGTCTATTAATTGTT
GCCGGGAAGCTAGAGTAAGTAGTTCGCCAGTTAATAGTTTGCGCAACGTTGTTGCCATTGCTACAGGCATCGTGGTG
TCACGCTCGTCGTTTGGTATGGCTTCATTCAGCTCCGGTTCCCAACGATCAAGGCGAGTTACATGATCCCCCATGTT
GTGCAAAAAAGCGGTTAGCTCCTTCGGTCCTCCGATCGTTGTCAGAAGTAAGTTGGCCGCAGTGTTATCACTCATGG
TTATGGCAGCACTGCATAATTCTCTTACTGTCATGCCATCCGTAAGATGCTTTTCTGTGACTGGTGAGTACTCAACC
AAGTCATTCTGAGAATAGTGTATGCGGCGACCGAGTTGCTCTTGCCCGGCGTCAATACGGGATAATACCGCGCCACA
TAGCAGAACTTTAAAAGTGCTCATCATTGGAAAACGTTCTTCGGGGCGAAAACTCTCAAGGATCTTACCGCTGTTGA
GATCCAGTTCGATGTAACCCACTCGTGCACCCAACTGATCTTCAGCATCTTTTACTTTCACCAGCGTTTCTGGGTGA
GCAAAAACAGGAAGGCAAAATGCCGCAAAAAAGGGAATAAGGGCGACACGGAAATGTTGAATACTCATACTCTTCCT
TTTTCAATATTATTGAAGCATTTATCAGGGTTATTGTCTCATGAGCGGATACATATTTGAATGTATTTAGAAAAATA
AACAAATAGGGGTTCCGCGCACATTTCCCCGAAAAGTGCCACCTGACGTCTAAGAAACCATTATTATCATGACATTA
ACCTATAAAAATAGGCGTATCACGAGGCCCTTTCGTCTCGCGCGTTTCGGTGATGACGGTGAAAACCTCTGACACAT
GCAGCTCCCGGAGACGGTCACAGCTTGTCTGTAAGCGGATGCCGGGAGCAGACAAGCCCGTCAGGGCGCGTCAGCGG
GTGTTGGCGGGTGTCGGGGCTGGCTTAACTATGCGGCATCAGAGCAGATTGTACTGAGAGTGCACCATATGCGGTGT
GAAATACCGCACAGATGCGTAAGGAGAAAATACCGCATCAGGCGCCATTCGCCATTCAGGCTGCGCAACTGTTGGGA
AGGGCGATCGGTGCGGGCCTCTTCGCTATTACGCCAGCTGGCGAAAGGGGGATGTGCTGCAAGGCGATTAAGTTGGG
TAACGCCAGGGTTTTCCCAGTCACGACGTTGTAAAACGACGGCCAGTGCCAAGCTTtttGCGGCCGC
