# Supplementary material for: CRISPR-Cas9 Gene Editing of the Sal1 Gene Family in Wheat
Source: Plants (Basel). 2022 Aug 30;11(17):2259. doi: 10.3390/plants11172259 (PMC9460255; doi:10.3390/plants11172259)
Supplement: Supplementary file 1 [file plants-11-02259-s001.zip › Mohr Figure S5 revised.pdf]

Figure S5A

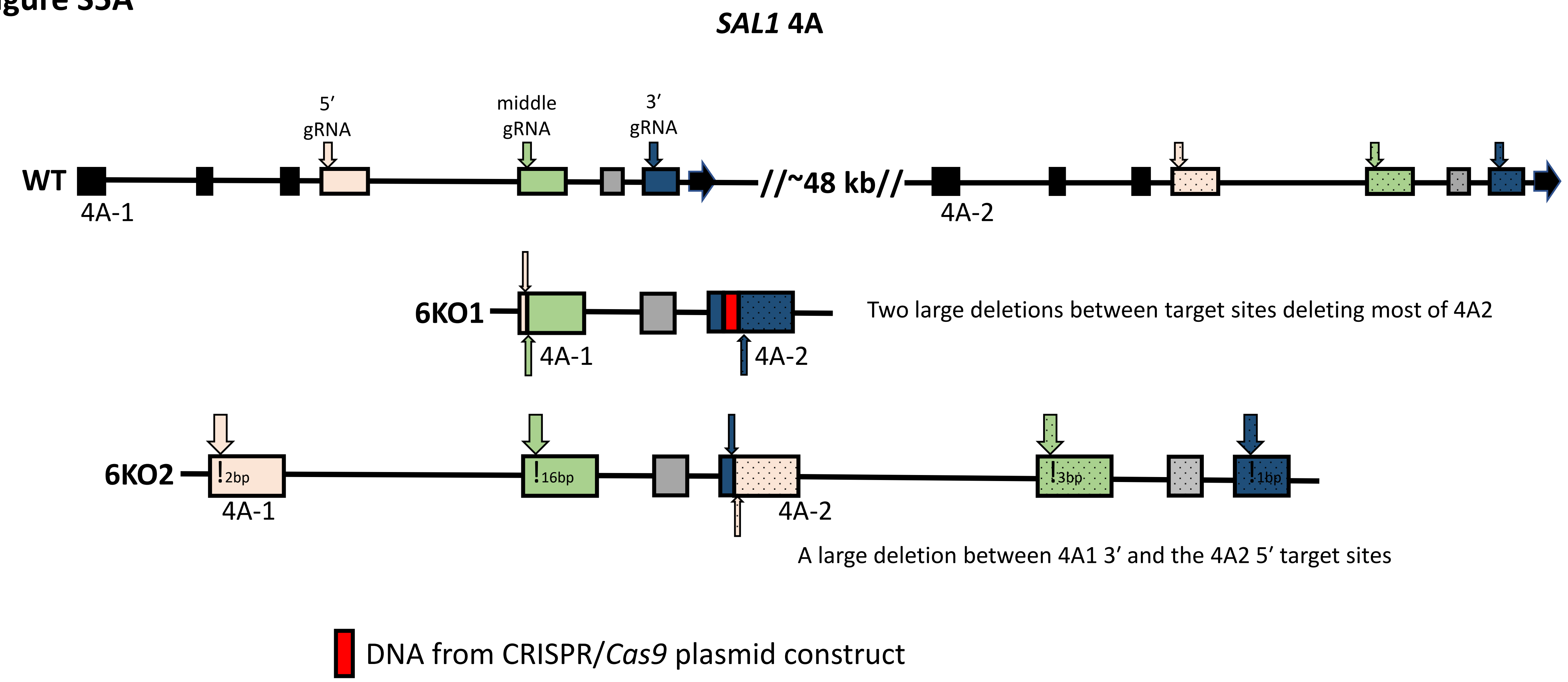

Figure S5: Major rearrangements, insertions, and deletions seen in 6KO1 and 6KO2 *sal1* mutants. The genes are shown as diagrams with boxes denoting exons and lines representing introns. The gRNA targeted regions are marked with the 3 colored arrows. The exons are also colored to aid in distinguishing one exon from another. The 4A2 exons are stippled to distinguish them from the 4A1 exons. The wildtype (WT) Bobwhite *Sal1* genotype is shown on the top of each panel with diagrammatic representations of the mutant genotype shown below. Regions of sequence inserted from the plasmid transformation construct are shown as red boxes. Small deletions are represented with an exclamation mark and label showing the number of deleted nucleotides. **A**. 4A1 and 4A-2 **B**. 7A **C**. 5D. For more information on the mutant genotypes see Table S5.

Figure S5B

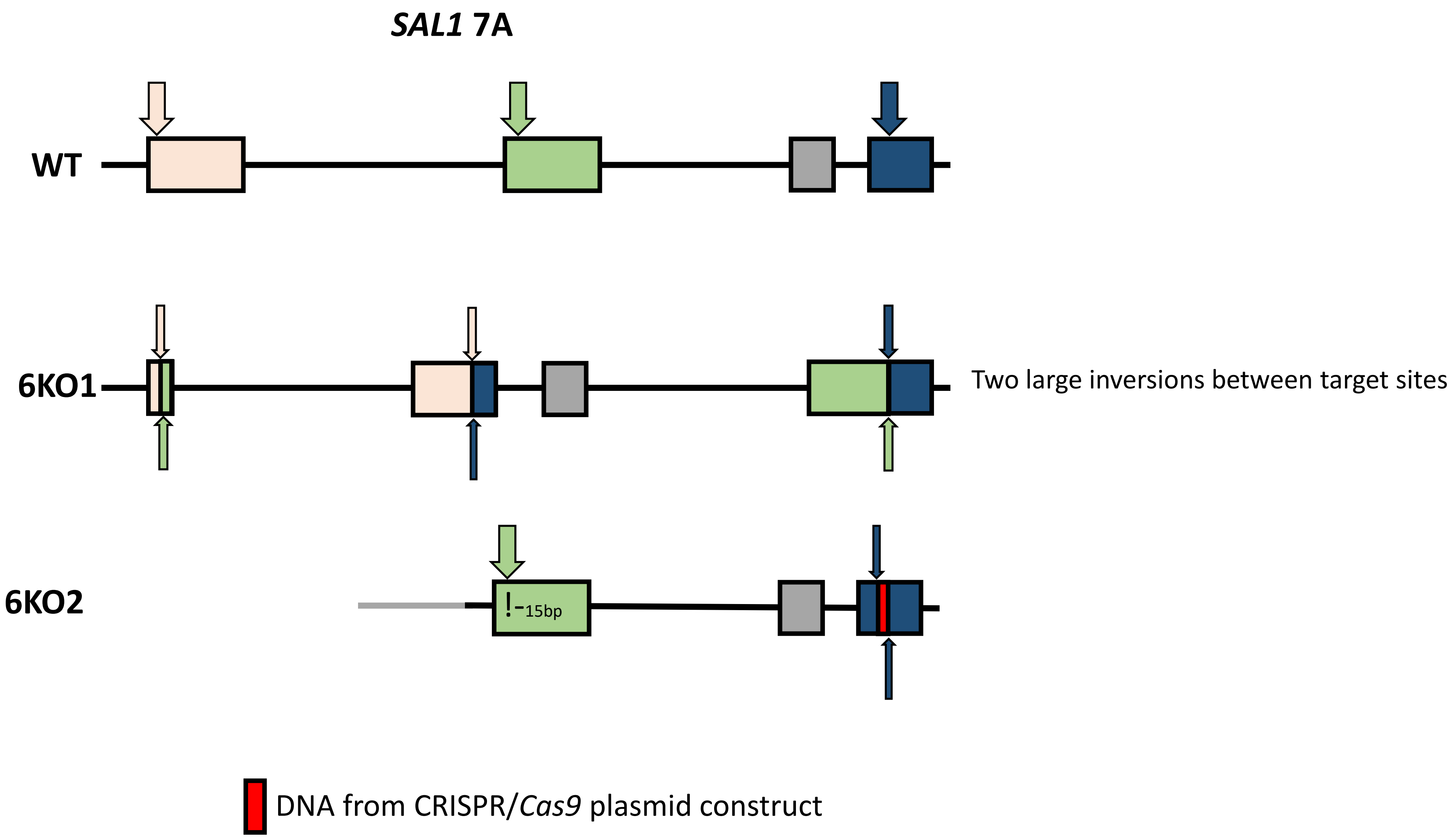

SAL1 7A
WT
6KO1
Two large inversions between target sites
6KO2
!-15bp
DNA from CRISPR/*Cas9* plasmid construct

Figure S5C

SAL1 5D

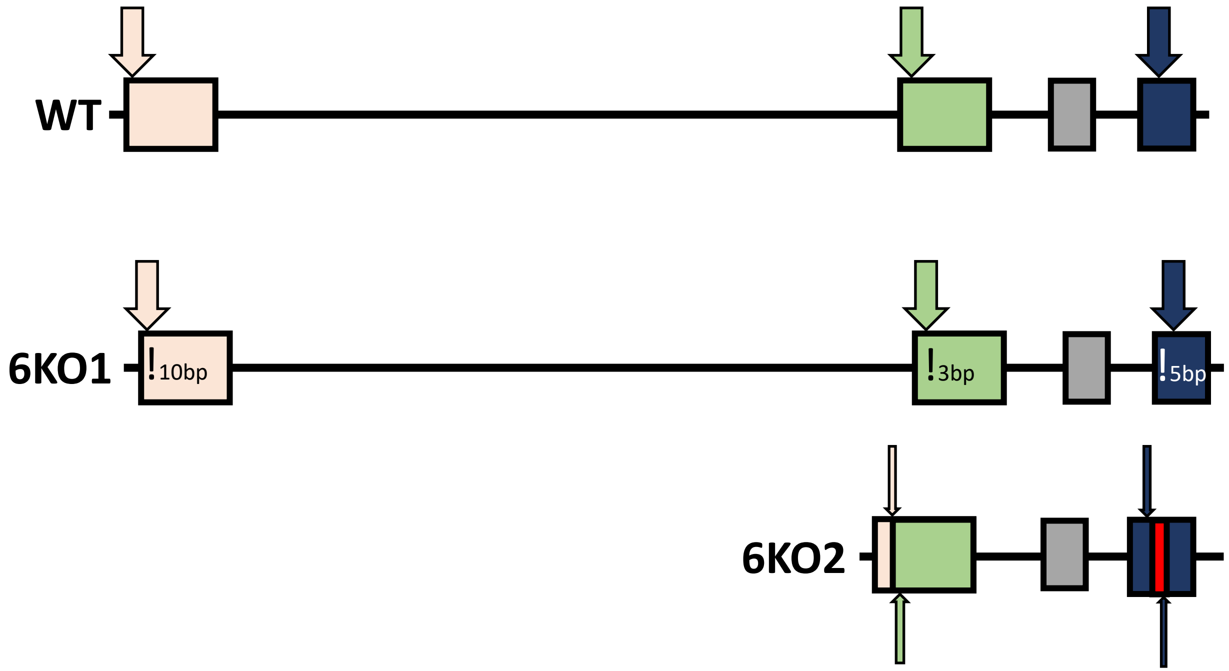

A large deletion between the 5’ and middle target sites

DNA from CRISPR/*Cas9* plasmid construct
