# Supplementary material for: CRISPR-Cas9 Gene Editing of the Sal1 Gene Family in Wheat
Source: Plants (Basel). 2022 Aug 30;11(17):2259. doi: 10.3390/plants11172259 (PMC9460255; doi:10.3390/plants11172259)
Supplement: Supplementary file 1 [file plants-11-02259-s001.zip › Mohr Figure S6 revised.pdf]

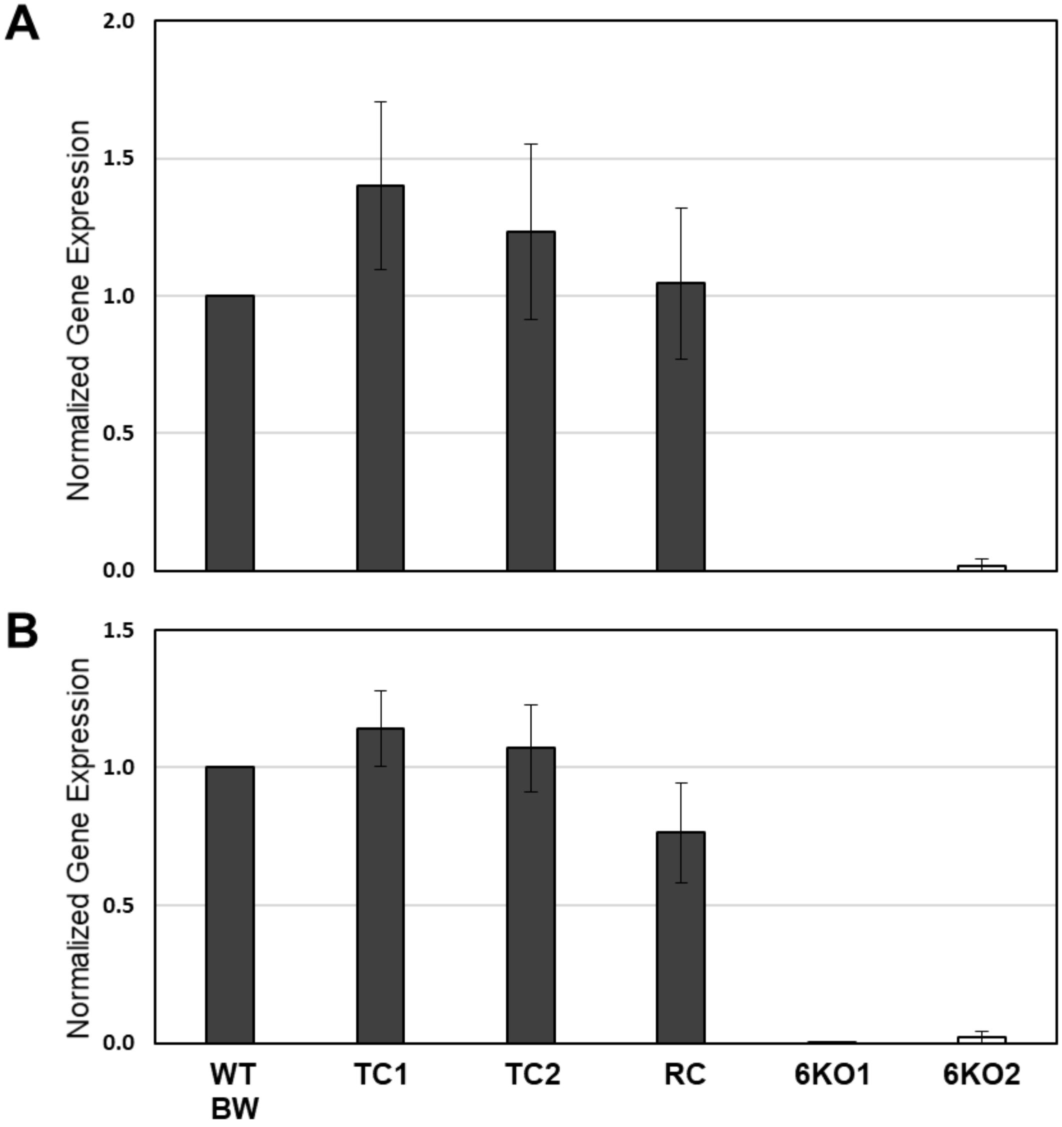

C

Primer	Oligo Sequence 5' to 3'	Product size (bp)	Target	Source
CJ705892_Forward1	GCCTCAGTGGTAGGAGCATT	116	CJ705892	[45]
CJ705892_Reverse1	TTCAGCAAATGCGGTGGTTG			
1491	AGTTGTTTTGGGCGTGTTGG	98	*Sal1* Chromosome 5	This study
1492	TCTACTTCAGCACCACAACCA			
1457	TGCGTGATTTTACTGGCTGTG	187	*Sal1* Chromosomes 7 and 4	This study
1458	GGCTGCATGATCCCATACCG			

Figure S6. *Sal1* transcript levels in the 6KO1 and 6KO2 mutants. Quantitative RT-PCR was used to examine *Sal1* transcript levels in wildtype Bobwhite (WT BW), other control lines (TC1, TC2, RC) and the 6KO1 and 6KO2 mutants. **A.** Normalized *Sal1* 5B and 5D transcript levels of control lines (grey bars) and the 6KO mutants (white bars). **B.** Normalized *Sal1* 7A, 7D, 4A1 and 4A2 transcript levels for of control lines (grey bars) and the 6KO mutants (white bars). **C.** The primers used for the reference transcript (CJ7052892) and *Sal1* transcripts.
