# Supplementary material for: CRISPR-Cas9 Gene Editing of the Sal1 Gene Family in Wheat
Source: Plants (Basel). 2022 Aug 30;11(17):2259. doi: 10.3390/plants11172259 (PMC9460255; doi:10.3390/plants11172259)
Supplement: Supplementary file 1 [file plants-11-02259-s001.zip › Mohr Figure S7 revised.pdf]

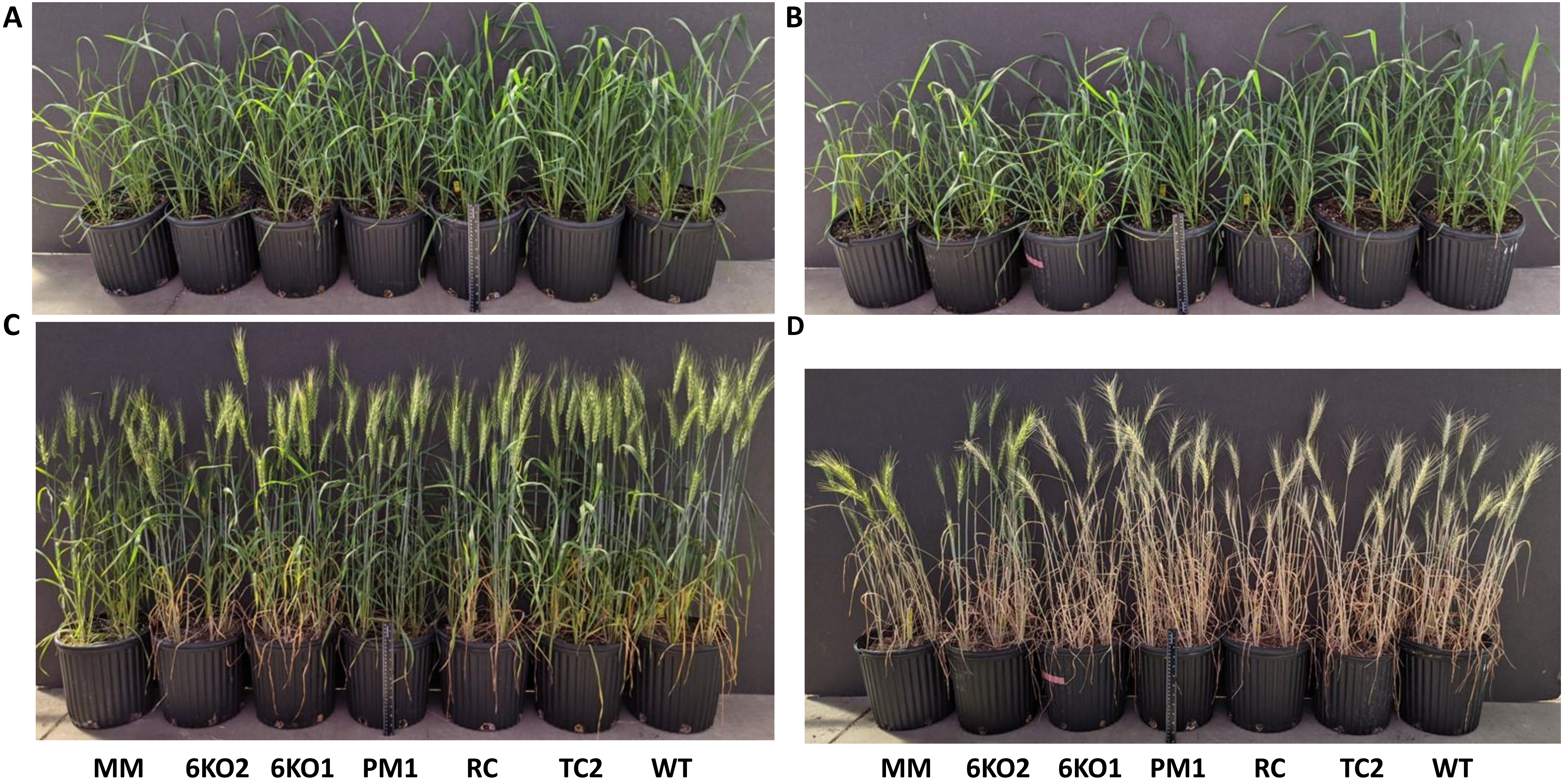


**Figure S7.** Growth of well-watered and drought treated *Sal1* lines in single pots **A**: Well-watered wheat lines 39 days after planting. **B**: Drought-treated wheat lines 39 days after planting (at the start of terminal drought). **C**: Well-watered wheat lines 56 days after planting. **D**: Drought treated plants at 56 days after planting (17 days after onset of drought).
