# Supplementary material for: CRISPR-Cas9 Gene Editing of the Sal1 Gene Family in Wheat
Source: Plants (Basel). 2022 Aug 30;11(17):2259. doi: 10.3390/plants11172259 (PMC9460255; doi:10.3390/plants11172259)
Supplement: Supplementary file 1 [file plants-11-02259-s001.zip › Mohr Figure S8 revised.pdf]

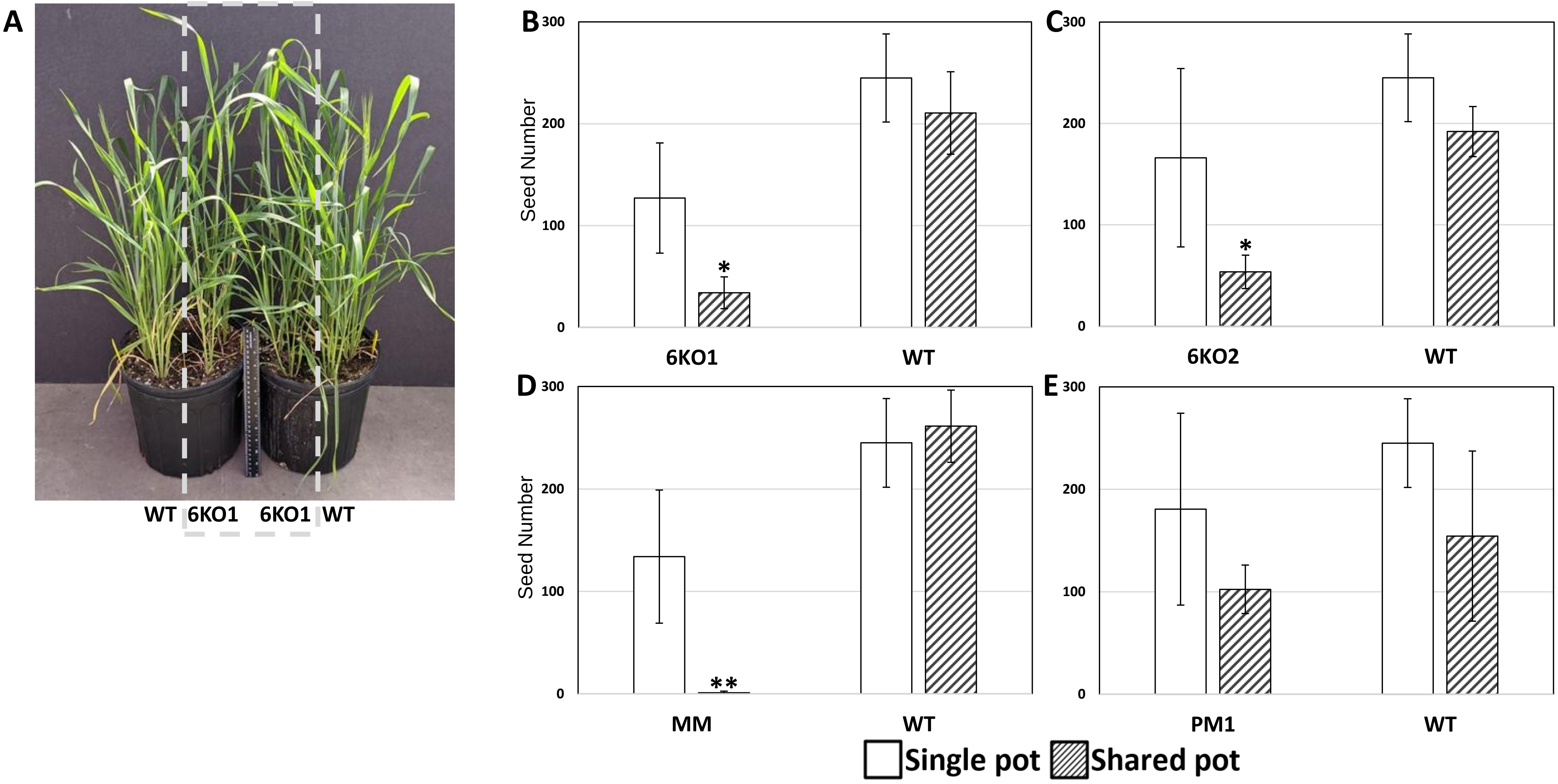

Figure S8. Growth and average seed number of well-watered *sal1* mutant lines in single and shared pots. **A**. Image of well-watered wildtype (WT) and 6KO1 plants in shared pots. The Bobwhite plants are along the outside edges of the pots shown, while the 6KO1 mutant plants are within the dashed box near the ruler. The average number of seed per plant for (**B**) 6K01 (**C**) 6KO2 (**D**) MM (**E**) PM1 in single (open bars) and shared pots (hashed bars) with wildtype plants. *=P<0.05; **= P<0.01; n≥3.
