# Supplementary material for: CRISPR-Cas9 Gene Editing of the Sal1 Gene Family in Wheat
Source: Plants (Basel). 2022 Aug 30;11(17):2259. doi: 10.3390/plants11172259 (PMC9460255; doi:10.3390/plants11172259)
Supplement: Supplementary file 1 [file plants-11-02259-s001.zip › Mohr Figure S9 revised.pdf]

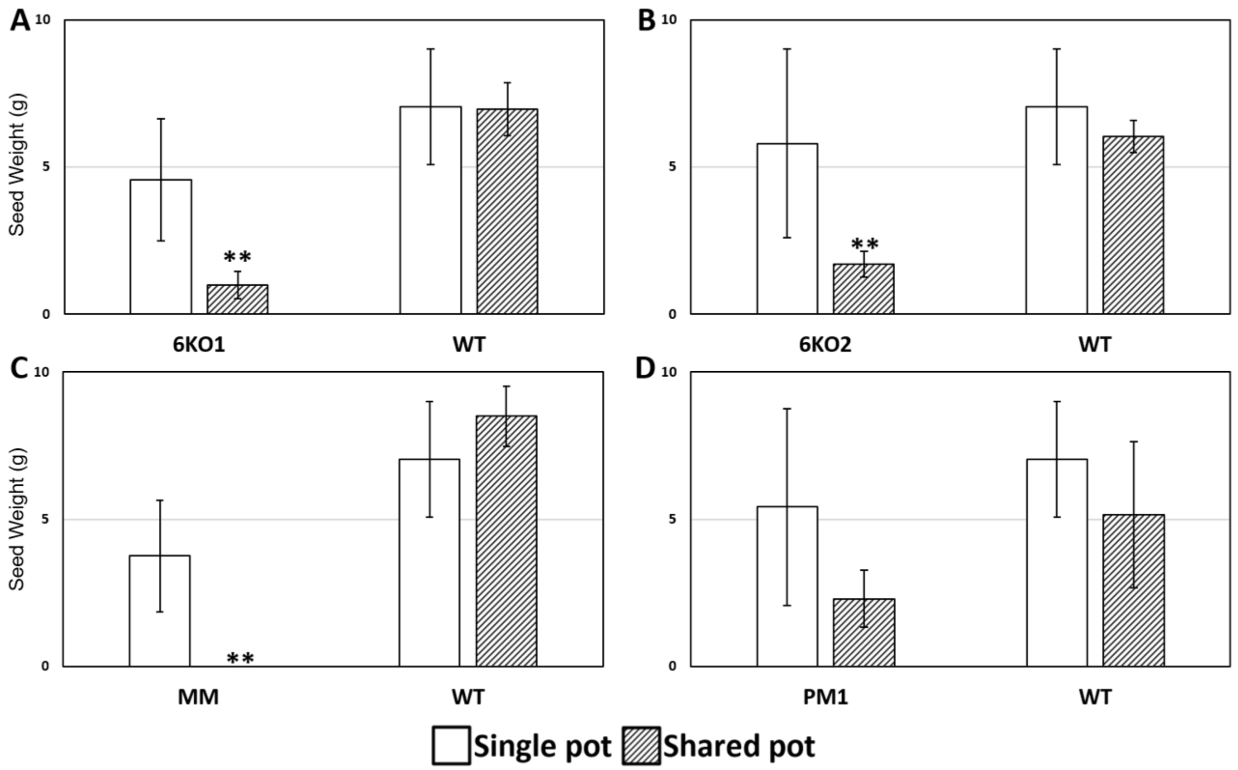

Figure S9. Average seed weight of well-watered *Sal1* mutant lines in single and shared pots compared to wildtype Bobwhite (**WT BW**). (**A**) 6K01 (**B**) 6KO2 (**C**) MM (**D**) PM1. **= P<0.01, n≥3.
